# Synthesis and Computational Evaluation of *N*‑Acetyl-Derived Schiff Bases Incorporating 1,2,4-Triazoles for Dual Inhibition of Prostate Cancer Cells and Carbonic Anhydrases

**DOI:** 10.1021/acsomega.5c03271

**Published:** 2025-08-22

**Authors:** Hilal Medetalibeyoğlu, Abdulmelik Aytatlı, Sevda Manap, Abdurrahman Atalay, Ahmet Buğra Ortaakarsu, Burak Tüzün, Parham Taslimi, Özlem Gürsoy-Kol, Ömer Faruk Karataş, Haydar Yüksek

**Affiliations:** † Department of Chemistry, Faculty of Arts and Sciences, 52975 Kafkas University, 36040 Kars, Turkey; ‡ Molecular Biology and Genetics Department, 226840 Erzurum Technical University, 25100 Erzurum, Turkey; § High Technology Application and Research Center, Erzurum Technical University, 25100 Erzurum, Turkey; ∥ Department of Nutrition and Dietetics, Faculty of Health Science, Avrasya University, 61080 Trabzon, Turkey; ⊥ Department of Chemistry, Faculty of Arts and Sciences, 37511 Gazi University, 06500 Ankara, Turkey; # Plant and Animal Production Department, Sivas Technical Sciences Vocational School, Sivas Cumhuriyet University, 58140 Sivas, Turkey; ∇ Department of Biotechnology, Faculty of Science, Bartin University, 74100 Bartin, Turkey; ○ Department of Chemistry, Faculty of Science, Karadeniz Technical University, 61080 Trabzon, Turkey

## Abstract

In this study, we synthesized a series of novel *N*-acetyl Schiff bases (**6a**–**e**) containing 1,2,4-triazole moiety and evaluated their potential as anticancer agents through both experimental and computational approaches. Cytotoxicity assays on prostate cancer (PC) (DU145) and normal epithelial cells (PNT1a) demonstrated selective inhibition, particularly for compounds **6a**, **6d**, and **6e**, with IC_50_ values of 73.25, 49.80, and 111.73 μM, respectively, in DU145 cells. Notably, **6d** exhibited a 10-fold selectivity toward cancer cells over normal cells. Enzyme inhibition studies demonstrated that compound **6d** exhibited the most potent inhibitory activity against the carbonic anhydrase isoforms hCAI and hCAII, with the lowest recorded IC_50_ and *K*_i_ values (7.12 and 9.26 μM for hCAI, and 10.62 and 11.72 μM for hCA II, respectively), suggesting strong potential for antiglaucoma therapeutic application. To elucidate molecular interactions, QM/MM molecular docking highlighted the strong affinity of compound **6d** for the active sites of CYP17A1, hCAI, and hCAII enzymes. The coordination of functional groups with key residues, particularly the Zn^2+^ ion and HEM group, was confirmed by detailed binding analyses. Molecular dynamics simulations further validated the stability of these interactions over a 100 ns trajectory, with **6d** maintaining robust engagement with the protein targets. This stability was reflected in consistent RMSD and RMSF profiles, with minimal fluctuations, particularly in CYP17A1 complexes, suggesting a stable binding conformation. The Markov State Model (MSM) analysis, integrated with TICA-FES and MM-GBSA calculations, revealed rapid conformational stabilization of **6d**, especially in CYP17A1 complexes. The observed deeper energy wells in diffusion maps indicate stronger binding affinities and reduced conformational transitions compared to reference inhibitors, such as abiraterone and acetazolamide. These computational insights align with experimental findings, suggesting that **6d** holds significant promise as a potent dual-target inhibitor with applications in prostate cancer therapy and glaucoma treatment.

## Graphical abstract

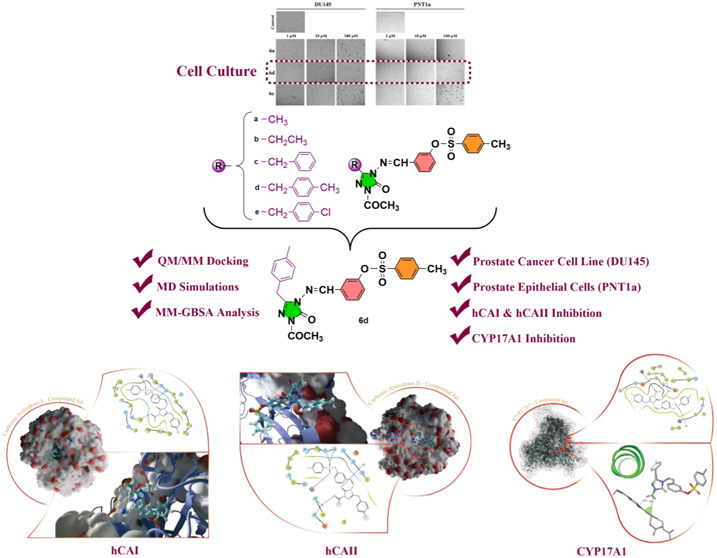

## 1 Introduction

Prostate cancer (PC) is the second most commonly diagnosed neoplasm in males worldwide, with 1,466,680 new cases and 396,792 deaths in 2022.^1^ Age, ethnicity, and genetic factors are considered among the risk factors for prostate cancer development and progression.^2–5^ Radiation therapy, chemotherapy, surgical methods, and hormonal therapy are the main treatment strategies in the management of prostate cancer.^6–8^

Androgen deprivation therapy (ADT) is the most widely employed treatment strategy for the PC, a condition that is not only life-threatening but also substantially diminishes patients’ quality of life.^9^ The primary goal of ADT is to block androgen receptors from promoting the expression of genes that drive cell proliferation.^9,10^ Additionally, the activation of pathways that enhance cellular survival and suppress apoptosis via androgen receptor activity provides strong justification for utilizing ADT in treating PC.^11,12^ During ADT, serum androgen levels are minimized, with two key enzymes in the adrenal gland—CYP11A1 and CYP17A1—being responsible for producing minor quantities of serum androgens through the biosynthesis of dehydroepiandrosterone and androstenedione.^13,14^ In advanced stages of PC, however, cancer cells can independently initiate androgen synthesis. At this juncture, the CYP17A1 enzyme becomes critically important, influenced by mechanisms such as epigenetic alterations, genetic mutations, gene amplification, and microRNA regulation.^15^ Given the capacity of the CYP17A1 enzyme to adapt beyond the scope of current therapeutic approaches, it becomes clear that ADT alone is insufficient in advanced PC cases.^16,17^ This highlights an urgent need for the development of new pharmacological agents and therapeutic strategies that can effectively target cancer cells and inhibit the autonomous production of androgens.

The acidic tumor microenvironment is a critical factor complicating the treatment of the PC.^18^ Research has shown that this acidic milieu significantly enhances the aggressive behavior of cancer cells, leading to rapid cell proliferation, increased metastatic potential, and accelerated tumor progression.^19^ This environment also interferes with immune system function, making it difficult for immune cells to mount an effective antitumor response. As a result, the presence of acidity in the tumor microenvironment is associated with increased therapeutic resistance and lower patient survival rates.^20^ Central to the maintenance of this acidic microenvironment are the enzymes carbonic anhydrase (CA) isoforms I and II, which play crucial roles in pH regulation.^21,22^ These enzymes facilitate the conversion of carbon dioxide (CO_2_) into bicarbonate (HCO_3_^–^) and protons (H^+^), thereby maintaining the delicate balance between intracellular and extracellular pH.^23,24^ From the tiniest microbes to the most intricate human tissues, these enzymes are present everywhere and significantly influence physiological processes that are essential for existence. As of right now, there are eight genetically different families of CA: α, β, γ, δ, ζ, η, θ, and ι. Only the α-class, known as human carbonic anhydrases (hCAs), has been found to exist in humans. There are 15 distinct isoforms of hCAs or CA-related proteins in the human body alone, and each one has developed specifically for a certain function in different tissues and cellular compartments. Several of these 15 isoforms—two mitochondrial isoforms, hCA VA and VB; five cytosolic isoforms, hCA I, II, III, VII, and XIII; four membrane-bound isoforms, hCA IV, IX, XII, and XIV; and a secreted hCA VI isoform—are catalytically active. These hCA isoforms’ proper expression and regulated activity are unquestionably necessary for a number of physiological functions. However, because of their overexpression or dysregulated activity, which results in a variety of disorders, their involvement in a number of pathological processes is also well-known. For example, illnesses as diverse as cerebral edema, glaucoma, and even the mysterious consequences of epilepsy and altitude sickness are linked to overexpression of hCA I and II.^25–28^ Targeting these enzymes may disrupt the cancer cells’ ability to maintain acidic conditions, thereby slowing tumor growth and metastasis. Recent studies suggest that optimizing the inhibition of CA activity at a physiological pH of 7.4 may enhance the efficacy of pharmacological agents.^29^ By restoring a more favorable pH for cellular metabolic processes, this approach could potentially yield a synergistic effect, improving the overall effectiveness of cancer therapies.

Building upon the previously discussed insights, we have designed a novel series of compounds (**6a**–**e**) leveraging the electronic properties of the 1,2,4-triazole moiety in Scheme 1. By optimizing the electronic transitions associated with the attached carbonyl group, our objective was to enhance therapeutic effects specifically in pancreatic cancer tissues. To further refine our approach, we evaluated the inhibitory potential of these compounds on the CYP17A1 enzyme, which is critical for androgen synthesis and is implicated in advanced stages of PC. This involved identifying substituents that would effectively interact with the active site of CYP17A1, based on electronic properties that strengthen coordination between HEM iron and carbonyl oxygen.

**Scheme 1:**
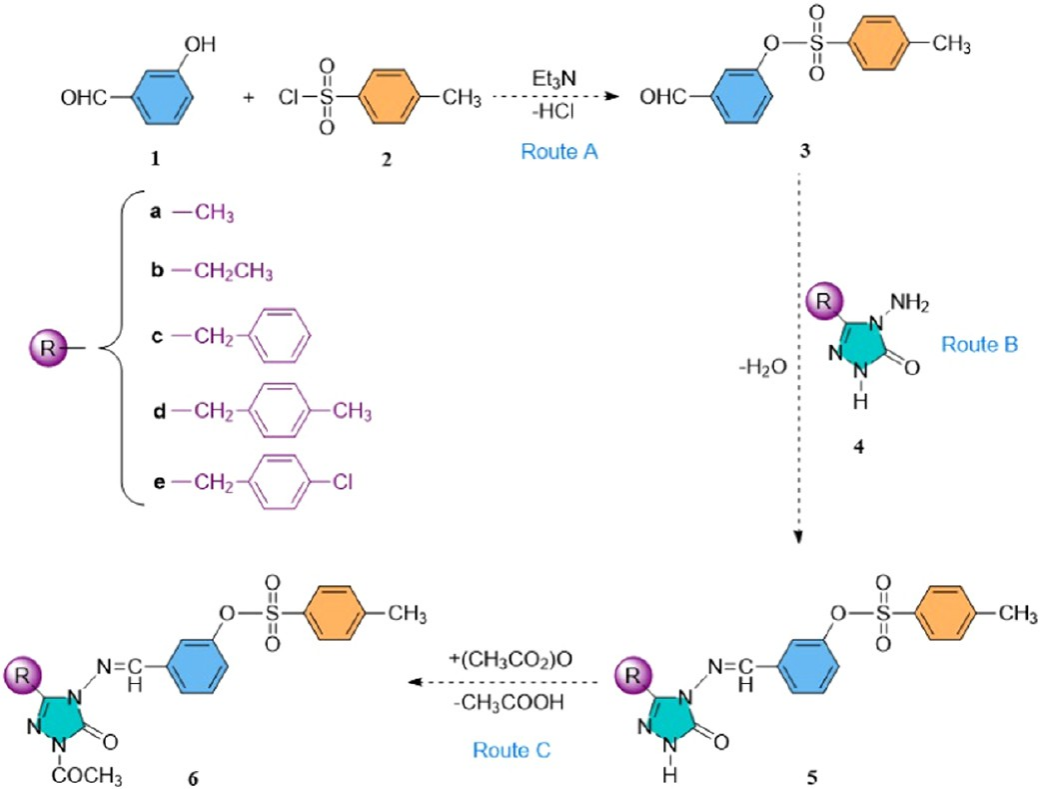
Synthesis Routes of the Compounds (**5a**–**e** and **6a**–**e**) (Routes A, B,^35^ and C)

A key component of our research involved cell culture experiments using the human prostate cancer cell line (DU145) to test the efficacy of these compounds against cancer cells. In parallel, selectivity tests were conducted on the human prostate epithelial cell line (PNT1a), representing healthy epithelial tissue, to assess the differential impact on noncancerous cells. The influence of our compounds on the tumor microenvironment was further evaluated by measuring their effect on CAI and II enzyme activities *in vitro*.

Integrating data from the literature with the strategic use of standardized functional groups and conformational constraints imposed by the imine group, we conducted extensive experimental analyses. Computer-aided simulations provided deeper insights into the underlying mechanisms of action. Advanced computational techniques, including quantum mechanical calculations, were employed to elucidate the interaction mechanisms of our most promising compounds with their molecular targets.

In the final phase of our research, state-of-the-art molecular dynamics simulations were utilized. We incorporated diffusion maps^30,31^ combined with free energy profiles and Markov State Models (MSM)^32–34^ to analyze transition rates between key molecular events. This approach facilitated a deeper understanding of how these compounds modulate the tumor microenvironment, particularly by targeting CAI and II to regulate pH levels.

## 2 Material and Methods

### 2.1 Experimental Details

Aldrich, Fluka, and Merck AG supplied chemicals used in the synthesis. The melting points of compounds **6a**–**e** were determined using the Electrothermal Melting-point Apparatus in open capillary tubes. The infrared spectra of compounds **6a**–**e** were documented via the PerkinElmer Instruments Spectrum one Fourier transform infrared (FT-IR) spectrophotometer. The proton/carbon-NMR spectra were analyzed with the assistance of the Bruker Ultrashield spectrophotometer at 400 MHz/100 MHz in deuterated dimethyl sulfoxide (DMSO-*d*_6_) utilizing tetramethylsilane (TMS). The Leco 932 Elemental Combustion System (CHNS-O) was used to conduct C, H, and N elemental studies.

#### 2.1.1 General Procedure for the Synthesis of 3-[((1-Acetyl-3-substituted-5-oxo-1,5-dihydro-4*H*-1,2,4-triazol-4-yl)-imino)-methyl]-phenyl-4-methylbenzenesulfonate (**6a**–**e**)

The five new compounds **6a**–**e** were obtained with high yields (85.71–65.71%) via the reaction of acetic anhydride and Schiff bases **5a**–**e**. The Schiff bases, 3-[((3-substituted-5-oxo-1,5-dihydro-4*H*-1,2,4-triazol-4-yl)-imino)-methyl]-phenyl-4-methylbenzenesulfonate (**5a**–**e**), were synthesized by the literature.^35^ The preparation process for 1-acetyl derivatives of 3-[((3-substituted-5-oxo-1,5-dihydro-4*H*-1,2,4-triazol-4-yl)-imino)-methyl]-phenyl-4-methylbenzenesulfonates (**6a**–**e**) was carried out as follows. A solution of 3-[((3-substituted-5-oxo-1,5-dihydro-4*H*-1,2,4-triazol-4-yl)-imino)-methyl]-phenyl-4-methylbenzenesulfonates (0.01 mol) was subjected to reflux with acetic anhydride (20 mL) for 0.5 h. The mixture underwent reflux for an additional 0.5 h after adding 100 mL of absolute ethanol. Through evaporation at a temperature range of 40–45 °C in vacuo and multiple recrystallizations of the residue from EtOH, pure forms of compounds **6a**–**e** were obtained as colorless crystals. Various methods were employed to confirm the structures of all compounds **6a**–**e**, including infrared (IR), proton-carbon (^1^H NMR and ^13^C NMR), nuclear magnetic resonance spectroscopy, and elemental analysis as depicted in Figures S1–15.

#### 2.1.2 3-[((1-Acetyl-3-methyl-5-oxo-1,5-dihydro-4*H*-1,2,4-triazol-4-yl)-imino)-methyl]-phenyl-4-methylbenzenesulfonate **(6a**)

Yield 3.96 g, (80.00%) mp. 131 °C. IR (KBr) υ_max_ 3082 (C=CH), 1726, 1699 (C=O), 1592 (C=N), 1373 and 1180 (SO) cm^–1^; ^1^H NMR (400 MHz, DMSO-*d*_6_, δ/ppm) δ 2.30 (s, 3H, CH_3_), 2.42 (s, 3H, PhCH_3_), 2.48 (s, 3H, COCH_3_), 7.19–7.22 (m, 1H, Ar–H), 7.48–7.52 (m, 5H, Ar–H), 7.55 (d, 2H, Ar–H; *J* = 8.40 Hz), 7.77–7.81 (m, 3H, Ar–H), 9.56 (s, 1H, N=CH); ^13^C NMR (100 MHz, DMSO-*d*_6_, δ/ppm) δ 11.06 (CH_3_), 21.15 (PhCH_3_), 23.37 (COCH_3_), [120.27 (CH), 125.28 (CH), 127.52 (CH), 128.26 (2CH), 130.28 (2CH), 130.85 (CH), 131.34 (C), 135.08 (C), 145.97 (C), 149.47 (C)] (Ar–C), 146.57 (Triazol C3), 147.71 (Triazol C5), 153.71 (N=CH), 165.95 (COO); Anal. Calcd for C_19_H_18_N_4_O_5_S (414.10): C, 56.06; H, 4.38; N, 13.52 found C, 55.93; H, 4.93; N, 13.05.

#### 2.1.3 3-[((1-Acetyl-3-ethyl-5-oxo-1,5-dihydro-4*H*-1,2,4-triazol-4-yl)-imino)-methyl]-phenyl-4-methylbenzenesulfonate (**6b**)

Yield 3.05 g, (85.71%) mp. 138 °C. IR (KBr) υ_max_ 3063 (C=CH), 1729, 1608 (C=O), 1573 (C=N), 1369 and 1183 (SO) cm^–1^; ^1^H NMR (400 MHz, DMSO-*d*_6_, δ/ppm) δ 1.23 (t, 3H, CH_2_CH_3_), 2.42 (s, 3H, PhCH_3_), 2.49 (s, 3H, COCH_3_), 2.69 (q, 2H, CH_2_CH_3_; *J* = 7.60 Hz), 7.21–7.23 (m, 1H, Ar–H), 7.48–7.56 (m, 4H, Ar–H), 7.76–7.81 (m, 3H, Ar–H), 9.57 (s, 1H, N=CH); ^13^C NMR (100 MHz, DMSO-*d*_6_, δ/ppm) δ 9.43 (CH_2_CH_3_), 18.50 (CH_2_CH_3_), 21.15 (PhCH_3_), 23.37 (COCH_3_), [120.11 (CH), 125.33 (CH), 127.60 (CH), 128.25 (2CH), 130.27 (2CH), 130.89 (CH), 131.32 (C), 135.09 (C), 145.96 (C), 149.49 (C)] (Ar–C), 147.95 (Triazole C3), 150.05 (Triazole C5); 153.72 (N=CH); 165.94 (COO); Anal. Calcd for C_20_H_20_N_4_O_5_S (428.12): C, 56.06; H, 4.70; N, 13.02 found C, 55.87; H, 4.23; N, 12.65.

#### 2.1.4 3-[((1-Acetyl-3-benzyl-5-oxo-1,5-dihydro-4*H*-1,2,4-triazol-4-yl)-imino)-methyl]-phenyl-4-methylbenzenesulfonate (**6c**)

Yield 3.22 g, (65.71%) mp. 144 °C. IR (KBr) υ_max_ 3059 (C=CH), 1730, 1608 (C=O), 1597 (C=N), 1368 and 1178 (SO) cm^–1^; ^1^H NMR (400 MHz, DMSO-*d*_6_, δ/ppm) δ 2.39 (s, 3H, PhCH_3_), 2.50 (s, 3H, COCH_3_), 4.09 (s, 2H, CH_2_Ph), 7.17–7.18 (m, 1H, Ar–H), 7.26–7.28 (m, 1H, Ar–H), 7.33–7.34 (m, 4H, Ar–H), 7.47 (d, 2H, Ar–H; *J* = 8.00 Hz), 7.49–7.55 (m, 2H, Ar–H), 7.78 (d, 2H, Ar–H; *J* = 8.00 Hz), 9.54 (s, 1H, N=CH); ^13^C NMR (100 MHz, DMSO-*d*_6_, δ/ppm) δ 9.43 (CH_2_CH_3_), 18.50 (CH_2_CH_3_), 21.13 (PhCH_3_), 23.45 (COCH_3_), 31.03 (CH_2_Ph), [119.94 (CH), 125.19 (CH), 126.76 (CH), 127.32 (CH),128.21 (2CH), 128.45 (2CH), 128.78 (2CH), 130.28 (2CH), 130.83 (CH), 131.37 (C), 134.52 (C), 135.08 (C), 145.96 (C), 149.46 (C)] (Ar–C), 147.87 (Triazol C3), 148.15 (Triazol C5), 153.53 (N=CH), 165.92 (COO); Anal. Calcd for C_20_H_22_N_4_O_5_S (490.13): C, 61.21; H, 4.52; N, 11.42 found C, 61.07; H, 4.23; N, 11.08.

#### 2.1.5 3-[((1-Acetyl-3-*p*-methylbenzyl-5-oxo-1,5-dihydro-4*H*-1,2,4-triazol-4-yl)-imino)-methyl]-phenyl-4-methylbenzenesulfonate (**6d**)

Yield 3.92 g, (75.38%) mp. 153 °C. IR (KBr) υ_max_ 3049 (C=CH), 1768, 1718 (C=O), 1593 (C=N), 1367 and 1181 (SO) cm^–1^; ^1^H NMR (400 MHz, DMSO-*d*_6_, δ/ppm) δ 2.26 (s, 3H, C_3_–PhCH_3_), 2.39 (s, 3H, PhCH_3_), 2.50 (s, 3H, COCH_3_), 4.04 (s, 2H, CH_2_Ph), 7.13 (m, 2H, Ar–H; *J* = 8.00 Hz), 7.15–7.18 (m, 1H, Ar–H), 7.22 (m, 2H, Ar–H; *J* = 8.00 Hz), 7.50–7.56 (m, 2H, Ar–H), 7.75–7.79 (d, 3H, ArH), 9.54 (s, 1H, N=CH); ^13^C NMR (100 MHz, DMSO-*d*_6_, δ/ppm) δ 9.43 (CH_2_CH_3_), 18.50 (CH_2_CH_3_), 21.61 (C_3_–PhCH_3_), 21.14 (PhCH_3_), 23.46 (COCH_3_), 30.64 (CH_2_Ph), [120.29 (CH), 125.18 (CH), 127.64 (CH), 128.22 (2CH), 128.65 (2CH), 129.09 (2CH), 130.29 (2CH), 130.84 (CH), 131.38 (C), 136.14 (C), 145.96 (C), 149.47 (C)] (Ar–C), 147.87 (Triazol C3), 148.30 (Triazol C5), 153.53 (N=CH), 165.93 (COO); Anal. Calcd for C_26_H_24_N_4_O_5_S (504.15): C, 61.89; H, 4.79; N, 11.10 found C, 61.41; H, 4.24; N, 10.78.

#### 2.1.6 3-[((1-Acetyl-3-*p*-chlorobenzyl-5-oxo-1,5-dihydro-4*H*-1,2,4-triazol-4-yl)-imino)-methyl]-phenyl-4-methylbenzenesulfonate (**6e**)

Yield 4.55 g, (84.28%) mp. 147 °C. IR (KBr) υ_max_ 3058 (C=CH), 1773, 1724 (C=O), 1570 (C=N), 1371 and 1184 (SO) cm^–1^; ^1^H NMR (400 MHz, DMSO-*d*_6_, δ/ppm) δ 2.39 (s, 3H, PhCH_3_), 2.49 (s, 3H, COCH_3_), 4.11 (s, 3H, CH_2_Ph), 7.15–7.18 (m, 1H, Ar–H), 7.36–7.41 (m, 4H, Ar–H), 7.48 (m, 2H, Ar–H; *J* = 8.00 Hz), 7.50–7.56 (d, 2H, Ar–H), 7.75–7.77 (m, 3H, Ar–H), 9.70 (s, 1H, N=CH); ^13^C NMR (100 MHz, DMSO-*d*_6_, δ/ppm) δ 21.14 (PhCH_3_), 23.45 (COCH_3_), 30.64 (CH_2_Ph), [120.38 (CH), 125.21 (CH), 127.59 (CH), 128.23 (2CH), 128.44 (2CH), 130.28 (2CH), 130.72 (2CH), 130.85 (CH), 131.34 (C), 130.84 (CH), 131.75­(C), 133.55 (C), 135.05 (C), 145.97 (C), 149.45 (C)] (Ar–C), 147.86 (Triazol C3), 149.45 (Triazol C5), 153.59 (N=CH), 165.91 (COO); Anal. Calcd for C_25_H_21_ClN_4_O_5_S (524.09): C, 57.20; H, 4.03; N, 10.67 found C, 57.13; H, 4.01; N, 10.38.

### 2.2 Cell Culture

In this research, the inhibitory effects of the compounds under investigation on cell viability were assessed using two distinct human cell lines: specifically, the DU145 cell line, representative of prostate cancer, and the PNT1a cell line, which serves as a model for normal human prostate epithelial cells. Cells were routinely cultured in RPMI-1640 medium supplemented with 10% fetal bovine serum, 1% penicillin/streptomycin (Gibco), and 1% l-glutamine. All cells were maintained in a 5% CO_2_ humidified incubator at 37 °C.

### 2.3 Cell Viability Assay

Effects of all compounds synthesized in this study on the viability of PNT1a and DU145 cells were analyzed using the Cell Viability Detection Kit-8 (CVDK-8, Ecotech Biotechnology, Erzurum, Turkey).^36^ In brief, cells were seeded at a concentration of 3 × 10^3^ cells per well within 100 μL medium in 96 well plates. Then, cells were treated with compounds at concentrations of 1, 10, and 100 μm for 24 h. After treatment with the test compounds, the medium was removed and fresh RPMI-1640 medium containing 1/10 CVDK- 8 reagent was added to each well, and plates were incubated for 3 h at 37 °C protected from light. After the incubation period, optical densities were measured using Epoch spectrophotometer at 450 nm. For cell culture, experiments were performed in triplicate, and data were analyzed by *t* tests.

### 2.4 Purification and Inhibition Investigations of hCA Isoenzymes

In this study, Sepharose-4B l-Tyrosine sulfanilamide affinity chromatography was employed to purify both CA isozymes in a single phase. A previous process was used to manufacture the column chemical material for affinity chromatography containing Sepharose-4B-l-Tyrosine-Sulfanilamide.^37^ As previously shown, the protein molecules’ flow in the column eluates was measured spectrophotometrically at 280 nm. To assess the inhibitory effects of new derivatives on CA isozymes, a spectrophotometric method similar to that described by Verpoorte et al. was applied.^38^ The substrate used, *p*-nitrophenyl acetate, was enzymatically converted by both isoenzymes into *p*-nitrophenolate ions. Changes in absorbance were measured at 348 nm at 3 min intervals throughout the investigation.^39,40^

### 2.5 Computational Details

To better understand the underlying mechanisms observed in our *in vitro* studies and to explore the protein–ligand interaction dynamics, a comprehensive set of *in silico* analyses was conducted. These computational approaches included molecular docking,^41,42^ molecular dynamics simulations, protein dynamics assessments,^5,43,44^ and Molecular Mechanics combined with Generalized Born and Surface Area Solvation (MM-GBSA)^45^ evaluations. All computations were primarily performed using the Maestro^46^ software from Schrödinger LLC, unless stated otherwise.

To further explore protein dynamics, we used advanced computational techniques such as Uniform Manifold Approximation and Projection (UMAP),^47^ Time-Lagged Independent Component Analysis (TICA)^48^ reduced free energy surface (FES) analysis,^49^ and Diffusion Map analysis integrated with MM-GBSA^50,51^ calculations. In these studies, we focused on compound **6d**, which demonstrated the highest activity in both cell culture and enzyme activation assays. For cell culture modeling, we utilized Human Cytochrome P450 CYP17A1, while for modeling of enzyme activity studies, we used the same carbonic anhydrase enzymes from the original *in vitro* experiments, along with reference compounds for comparison.

#### 2.5.1 Molecular Docking

The molecular docking approach was utilized to evaluate the binding affinities of compound **6d** to protein structures and to predict its potential interactions with target proteins. To ensure accurate localization of compound **6d** within these structures, techniques consistent with the induced fit model were applied, allowing for full flexibility of both the protein and ligand.

##### 2.5.1.1 Ligand Preparation

Upon completing the geometry optimization of compound **6d**, its quantum mechanical tautomeric and conformational states^52^ were systematically analyzed. Among the 55 conformations examined, the one with the lowest energy was identified and selected for further studies. These optimized conformations were subsequently processed for ionization at a physiological pH of 7.4, utilizing the LigPrep module^53^ integrated with Epik software.^54^ This step was carried out under the OPLS4 force field^55^ to ensure accurate parametrization. Additionally, the analysis accounted for potential isomerization states, with all possible isomers of compound **6d** being included in the ligand preparation process.^56,57^ The structure elucidation data guided the inclusion of these isomers to comprehensively represent the compound’s diverse structural forms.

##### 2.5.1.2 Preparation of Protein Structures

The study employed the human cytochrome P450 CYP17A1 crystal structure, identified by the Protein Data Bank (PDB) code 3RUK.^58^ This specific construct of CYP17A1 was chosen due to its inclusion of abiraterone as a cognate ligand,^59^ which is currently the only clinically approved inhibitor of CYP17A1 for prostate cancer therapy. For carbonic anhydrases CAI and CAII, the crystal structures with PDB codes 1AZM^60^ and 3HS4 ^61^ were selected, respectively. These structures were chosen because they contain acetazolamide, the reference compound used in *in vitro* experiments, as their cognate ligand. The selection criteria for all protein structures prioritized high-resolution data, specifically focusing on those with a resolution better than 3 Å, to ensure structural accuracy.

The structures downloaded in pdb format were surrounded by water molecules using HydraProt,^62^ which was trained using deep learning methods with the positions of 1,743,108 experimental water molecules in 4000 protein crystal structures in the PDB. These core systems were then preprocessed using the Protein Preparation Wizard^63^ in Maestro, including the formation of disulfide bonds and metal bonds (coordination), the addition of hydrogens and the determination of ionization states for pH 7.4. After optimization of hydrogen bonding for pH 7.4 using PROPKA,^64^ the OPLS4 force field^55^ was applied. In the case of the detection of missing amino acids in protein structures, protein structure accuracy was ensured by making additions in the appropriate conformation.

##### 2.5.1.3 Ligand Docking and QM Docking

The prepared protein structures and compound **6d** were subjected to an initial docking procedure for each protein structure. The initial docking procedure, conducted using the Glide module,^65^ facilitated the identification of the **6d** conformation with the lowest energy potential, thereby enabling its potential replacement of cognate ligands within protein structures. In this phase, the grid preparation process for CYP17A1 was optimized. In the preparation of the grid, the partial charge cutoff value was set to 0.50, and the scaling factor was maintained at the default setting. In the context of ligand docking, the scaling factor was set to 0.90, and the partial charge cutoff was set to 0.50. These adjustments were made to prevent the neglect of these interactions, as close interactions will already be accounted for in the flexible docking step.^66^ The default settings were employed for CAI and CAII. The reason why optimized parameters are not needed is that the binding sites of carbonic anhydrase enzymes occupy a transitional area. In each instance of the ligand docking operation, the Extra Precision (XP) setting was selected, and 20 poses per ligand were requested. In this step, the force field is set to OPLS4. As the cognate ligands were employed as a reference for critical interactions and protein dynamics, a validation process was conducted to assess the accuracy of the initial docking step. This validation process is shown in Figures S16–S18.

Following the initial docking stage, during which the ligand was treated as flexible and the protein structure as rigid, a fully flexible docking approach was employed.^67^ The objective of this phase is to create an accurate model of the ligand and protein structure with respect to each other, in accordance with the Induced Fit Model.^68–71^ To this end, redocking was conducted, and the conformation space of **6d** was reassessed by requesting 80 poses in the extended sampling setting. In accordance with the design of the ligands, the positions of the metal atoms were entered into the software as parameters in order to elaborate the coordination with metal atoms in detail. The objective is to create realistic protein–ligand complexes that account for interactions with metal atoms.^72^

The Qsite^73^ application, utilizing the Jaguar module,^74^ was employed to examine the alterations in electron configuration and polarity resulting from the influence of the protein structure in **6d**, which was subjected to the protein structure.^75^ The ligand was selected for quantum mechanical calculations, and the protein structure was treated with the OPLS4 force field. In order to perform DFT calculations on **6d** embedded in CYP17A1 enzyme, it was deemed preferable to utilize the M05 functional^76^ and LACVP++** basis set,^77,78^ given that they are known to yield realistic results in biomolecules.^79^ In the case of **6d** embedded in carbonic anhydrase enzymes, the LACVP* basis set was employed in conjunction with the B3LYP functional.^79,80^ The rationale behind the use of different methods and basis sets for two different biomolecules in quantum calculations is that the electronic structures and energy levels of these molecules are different, so we tried to select the most appropriate calculation methods and basis sets to obtain the most accurate energy state of each biomolecule.^81^

The electron configuration of **6d**, which undergoes alteration in protein structures, was removed from the protein structures and a single point energy calculation was performed without modification of its conformation. It was thus determined what kind of electron configuration the compound has in this conformation in the absence of interaction. A comparison was made between **6d** in the protein structure and **6d** in the same conformation in order to elucidate the impact of the protein structure on the ligand. The same methodology and basis sets utilized in QSite were employed in the single-point energy calculation with the Jaguar module.

#### 2.5.2 Molecular Dynamics

To assess the stability and interaction dynamics of the protein–ligand complexes, molecular dynamics (MD) simulations were carried out with high temporal resolution. These simulations aimed to replicate physiological conditions as closely as possible, thus allowing for a more realistic evaluation of the molecular docking outcomes.^82^ For a comparative assessment of **6d** against known reference compounds, simulations were also conducted using protein structures containing crystal data with the reference ligands. This enabled a direct comparison of the binding behavior and stability between **6d** and established ligands within the same experimental framework. By integrating these computational analyses, we sought to interpret and explain the results observed *in vitro* studies. The insights derived from the simulations provided a scientific rationale for the observed experimental effects, thereby enhancing our understanding of the molecular interactions underpinning the efficacy of **6d**.

##### 2.5.2.1 System Setup

The protein–ligand complexes were placed in a solvent box filled with SPC (simple point charge) water molecules, measuring 10 Å in each dimension.^83^ Unlike carbonic anhydrases, the enzyme CYP17A1 is anchored to the membrane of the endoplasmic reticulum.^58^ To study CYP17A1 in a context that closely mimics its native environment, a POPC (palmitoyl-oleoyl-phosphatidylcholine) membrane model^84^ was constructed. This membrane system was further enhanced by adding cholesterol and sphingolipids, reflecting the natural composition of the endoplasmic reticulum, which contains these components at much lower levels compared to the plasma membrane.^85,86^ As a result, only minimal amounts of these lipids were incorporated to align with the actual physiological conditions. To maintain electrostatic neutrality, sodium ions were added to balance the system based on its specific charge requirements. Additionally, a concentration of 0.15 M NaCl, mimicking physiological ionic strength, was introduced. The final system was prepared using the OPLS4 force field to optimize the accuracy of the molecular dynamics simulations.

##### 2.5.2.2 Molecular Dynamic System Protocols

To ensure the systems reflected the physiological environment, it was essential to maintain a constant number of particles, pressure, and temperature. To ensure the appropriate adjustment of these parameters, a constant number of particles was maintained by the provision of high-energy barriers around the system. A Nose-Hoover thermostat^87^ was employed to maintain a constant temperature of 300 K. A constant pressure of 1 bar was established using a Martyna-Tobias-Klein barostat.^88^ Simulations were conducted using the Desmond module^89^ in two phases. The first phase consisted of a 2 ps relaxation phase, followed by a 100 ns simulation generation phase, during which 1000 frames were requested.

#### 2.5.3 MM-GBSA Calculation

To evaluate the stability of the complexes formed by **6d** with the target proteins, free energy calculations were performed. The binding free energies for each frame throughout the molecular dynamics simulations were determined using the MM-GBSA method, and the results were subsequently visualized through graphical plots. For these calculations, the Prime module^90^ was utilized to implement the MM-GBSA approach. The simulations incorporated the VSGB solver model,^91^ with the OPLS4 force field employed to enhance the precision of the results. The computations followed a specific formula to ensure consistency and accuracy in estimating the free energies of binding across all sampled conformations.

$$\Delta G=E_{\mathrm{complex}}\left(\mathrm{minimized}\right)-\left[E_{\mathrm{ligand}}\left(\mathrm{minimized}\right)+E_{\mathrm{receptor}}\left(\mathrm{minimized}\right)\right]$$

#### 2.5.4 Analysis of Protein Dynamics and Effect of Ligand on Proteins

Upon completing the initial MD simulations, a detailed investigation was conducted to assess how both the reference compounds and **6d** influenced protein structures, induced conformational changes, and potentially affected physiological processes. To achieve this, a comprehensive analysis was performed using a range of advanced techniques. The MSM^92^ were utilized to capture the dynamic conformational states of the proteins. Additionally, dimensionality reduction through UMAP was employed to visualize structural changes. The influence of ligands on protein folding was further analyzed using TICA-FES^93^ with a lag time of 500 frames. To identify structural clusters, diffusion maps were generated, and clustering was performed using the OPTICS algorithm,^94^ with binding energy data obtained via MM-GBSA calculations. These analyses were conducted using custom Python scripts specifically developed for this study. The scripts are freely accessible and can be downloaded from the GitHub repository at: https://github.com/cannabinoid13/MDScripts.

## 3 Results and Discussion

### 3.1 Synthesis

Scheme 1 illustrates the synthetic pathways employed to obtain the compounds investigated in this study. In a previous study, our research team successfully synthesized compounds **5a**–**e** by reacting 3-formyl phenyl 4-methylbenzenesulfonate (**3**) with five different compounds (**4a**–**e**) under acetic acid conditions.^35^ In this current research, we explored the synthesis of five novel compounds, namely 3-[((1-acetyl-3-substituted-5-oxo-1,5-dihydro-4*H*-1,2,4-triazol-4-yl)-imino)-methyl]-phenyl-4-methylbenzenesulfonates (**6a**–**e**) through the reaction of Schiff bases **5a**–**e** with acetic anhydride in Scheme 1. The synthesis process yielded these compounds efficiently, with yields ranging from 85.71 to 65.71%. To verify the structures of the five novel compounds (**6a**–**e**), comprehensive analytical techniques such as infrared (IR) spectroscopy, proton-carbon (^1^H NMR and ^13^C NMR) spectroscopy, along with elemental analysis, were employed. The newly synthesized *N*-acetyl Schiff bases (**6a**–**e**) exhibited C=CH stretching vibrational bands in their IR spectra, observed within 3082–3049 cm^–1^ intervals. In the spectra, the C=O stretching vibrational bands have been observed within the 1773–1726 and 1724–1608 cm^–1^ ranges, while the C=N stretching vibrational bands were detected in the 1593–1570 cm^–1^ range. Additionally, the SO vibrational bands of the compounds (**6a**–**e**) were recorded within the 1371–1367 and 1184–1178 cm^–1^ intervals. In the ^1^H NMR spectrum, acidic NH proton signals were observed as approximately 12.00 ppm in Schiff bases (**5a-e**) registered in the literature,^35^ while these signals in *N*-type compounds (**6a**–**e**) disappeared. It was concluded that the *N*-acetyl Schiff bases had taken place based on the disappearance of NH signals. In addition, the N=CH proton signals are detected within the range of δ 9.70–9.54 ppm, while the COCH_3_ proton signals display between δ 2.50–2.49 ppm. Upon analysis of the ^13^C-NMR spectra, it was observed that the imine carbon signals corresponding to N=CH were detected within the range of δ 153.72–151.53 ppm. In addition, it was observed that the carbonyl signals of COCH_3_, which are bound to the triazole ring in the compounds **6a**–**e**, were detected at intervals ranging from 23.46 to 23.37 ppm.

### 3.2 Cell Culture

We evaluated the viability of prostate cancer and normal prostate epithelial cells using a CVDK-8 assay. Our results demonstrated that the compounds synthesized in this study inhibited the viability of cancer cells, as shown in Figure 1. Although **6c** and **6d** did not suppress the viability of cancer cells in low concentrations, they effectively reduced the number of cells exposed to those chemicals. Meanwhile, **6a** and **6e** decreased the viability of cancer cells in a dose-dependent manner in Figure 1.

**Figure 1:**
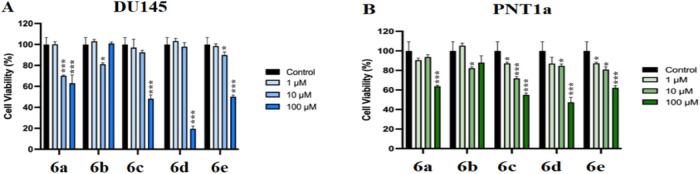
Effects of compounds **6a**–**e** on the viability of DU145 (A) and PNT1a (B) cells. Cells were treated with 1, 10, and 100 μM concentrations of each compound for a defined incubation period, and cell viability was assessed using a standard viability assay. Bars represent the mean ± standard deviation (SD) of triplicate experiments. Statistical significance was calculated relative to the control group: *p* < 0.05 (*), *p* < 0.01 (**), *p* < 0.001 (***).

Although all molecules affected the viability of normal prostate epithelial cells, the impacts of **6a**, **6d**, and **6e** were limited compared to prostate cancer cells, suggesting their selective targeting potential on cancer cells over normal cells in Figure 2 and Table 1. Therefore, they might be considered as putative antiprostate cancer agents with potentially lower side effects, which have to be evaluated with further *in vitro* and *in vivo* functional assays.

**Figure 2:**
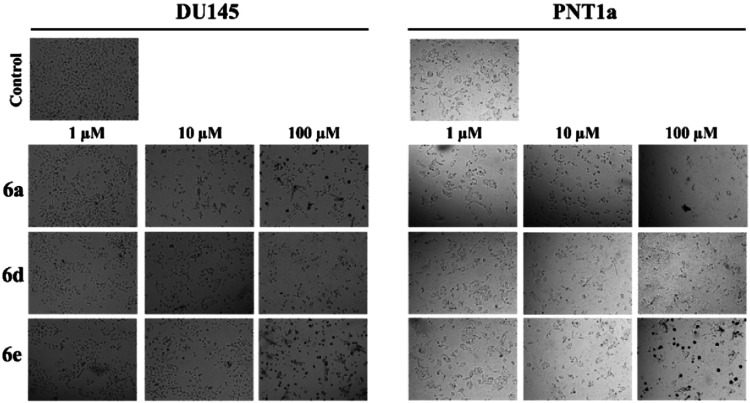
Visualization of DU145 and PNT1a cells exposed to varying doses of compounds **6a**, **6d**, and **6e**.

**Table 1:** IC_50_ Values of Compounds **6a**–**e**, Determined Based on the Viability of DU145 and PNT1a Cells Following 24 h Treatment

	IC_50_ (μM)		selectivity indices (SI)
compound	DU145	PNT1a	PNT1a IC_50_/DU145 IC_50_
**6a**	73.25 ± 11.54	493.59 ± 106.82	6.74
**6b**	N.A.	N.A.	--
**6c**	137.78 ± 17.79	167.53 ± 43.09	1.22
**6d**	49.80 ± 11.12	113.44 ± 27.07	2.28
**6e**	111.73 ± 9.78	584.60 ± 108.04	5.23

### 3.3 Enzyme Activity

The inhibitory effects of the novel compounds **6a**–**e** against hCAI are shown in Table 2. The compounds under investigation had some fairly interesting hCAI inhibition features. Overall, the novel compounds **6a**–**e** showed outstanding inhibitory activity, with *K*_i_ values ranging from 9.26 ± 0.88 to 16.72 ± 2.14 μM. Furthermore, acetazolamide, the traditional hCA inhibitor used in this study, displayed a *K*_i_ value for hCAI of 14.58 ± 0.91 μM. These results show that certain unique compounds exhibit much better hCAI and II suppression than newly produced compounds and standard drugs. Indeed, with *K*_i_ values of 9.26 ± 0.88, 13.12 ± 1.02, and 14.03 ± 1.15 μM, respectively, the compounds **6d**, **6c**, and **6a** demonstrated an effective inhibitory profile against hCAI in Table 2. The IC_50_ values of all novel studied compounds on hCAI exhibited the following order: **6d** (7.12 μM, *r* ^2^: 0.989) < **6c** (10.20 μM, *r* ^2^: 0.957) < **6a** (11.36 μM, *r* ^2^: 0.985) < **AZA** (12.17 μM, *r* ^2^: 0.949) < **6e** (12.33 μM, *r* ^2^: 0.950) < **6b** (13.67 μM, *r* ^2^: 0.926). Additionally, the new series of compounds (**6a**–**e**) for the hCAII enzyme had IC_50_ values ranging from 10.62 to 18.35 μM and *K*_i_ values ranging from 11.72 ± 1.01 to 21.87 ± 3.48 μM in Table 2. The results demonstrated that compared to acetazolamide (IC_50_: 16.58 μM), a CA inhibitor, all novel derivatives **6a–e** demonstrated more potent hCAII inhibitory activities. However, the highest effective *K*_i_ values, 11.72 ± 1.01, 17.31 ± 2.41, and 21.87 ± 3.48 μM, respectively, were produced by compounds **6d**, **6c**, and **6e**. A progressive neuropathic disorder of the eyes, glaucoma affects 80 million people globally. Based on the anatomic nature of the aqueous humor, this ocular ailment is classed as either open-angle or angle-closure glaucoma, with the latter being the most frequent. As glaucoma progresses, damage to the optic nerve fibers may cause blind spots. Glaucoma can result in lifelong blindness if left untreated. An eye drainage system issue may result in fluid buildup and elevated intraocular pressure (IOP), harming the optic nerve.^95,96^

**Table 2:** Enzyme Inhibition Results (in μM) of Novel Compounds against hCAI and II

	IC_50_ (μM)			*K*_i_ (μM)		
compounds	hCA I	*r* ^2^	hCA II	*r* ^2^	hCA I	hCA II
**6a**	11.36	0.985	15.33	0.926	14.03 ± 1.15	19.67 ± 2.48
**6b**	13.67	0.926	18.35	0.942	16.72 ± 2.14	21.87 ± 3.48
**6c**	10.20	0.957	14.50	0.968	13.12 ± 1.02	17.31 ± 2.41
**6d**	7.12	0.989	10.62	0.961	9.26 ± 0.88	11.72 ± 1.01
**6e**	12.33	0.950	16.78	0.958	14.15 ± 1.10	18.12 ± 2.47
**AZA** ^*^	12.17	0.949	16.58	0.970	14.58 ± 0.91	19.43 ± 3.28

The current method utilized in the clinic to treat open-angle glaucoma is lowering ocular IOP, either with medication or surgery. Specifically targeting IOP-lowering medications, six pharmacological classes, prostaglandin analogs, sympathomimetics, cholinomimetics, β-blockers, and rho-kinase inhibitors are clinically employed to rectify unusually elevated intraocular pressure. CAs are a common type of metalloenzyme that catalyzes the hydration process of CO_2_.^97,98^ In humans, α-class isoforms totaling 16 have been found. These isoforms have distinct tissues and subcellular localizations and react to different modulators in diverse ways. In glaucoma, there has been evidence of isoforms II and IV being overexpressed. By lowering the synthesis of bicarbonate and, in turn, the secretion of aqueous humor, CAIs can lower intraocular pressure. Therefore, there may be an antiglaucoma effect from developing selective CAIs of these isoforms.^99–102^

### 3.4 Computational Studies

Molecular docking analyses were independently carried out for the CAI, CAII, and CYP17A1 enzymes, indicating that **6d** displayed strong affinity toward each of these protein structures. To refine the binding interactions, the final conformations of the protein–ligand complexes were determined using a combined quantum mechanics/molecular mechanics (QM/MM) approach, as previously outlined. The detailed results of these docking studies are provided below.

#### 3.4.1 QM/MM Docking for CYP17A1, CAI, and CAII

Upon binding to the CYP17A1 protein, **6d** demonstrated a significant potential to establish a coordination bond between the carbonyl oxygen of its triazole moiety and the central iron atom within the HEM group of the enzyme. This binding is further stabilized by a network of primarily hydrophobic interactions between the ligand and the residues lining the protein’s binding pocket. Compound **6d** is deeply embedded within the active site, maintaining close contact with key amino acids surrounding the HEM group. Notably, the carbonyl oxygen of the triazole ring showed a strong propensity to form hydrogen bonds with the Val402 residue, mediated by bridging water molecules that penetrate into the active site. Additionally, the oxygen atom of the ketone group attached to the triazole ring coordinates directly with the iron center of the HEM, with an interaction distance measured at 2.41 Å. The aromatic ring extending from the triazole group, along with its methyl-substituted branch, is oriented at an angle of 60.9° relative to the HEM plane, positioning it toward a helical region of the protein. The detailed docking pose of **6d** within the binding pocket, along with the hydrophobic interactions that stabilize its orientation, are depicted in Figure 3.

**Figure 3:**
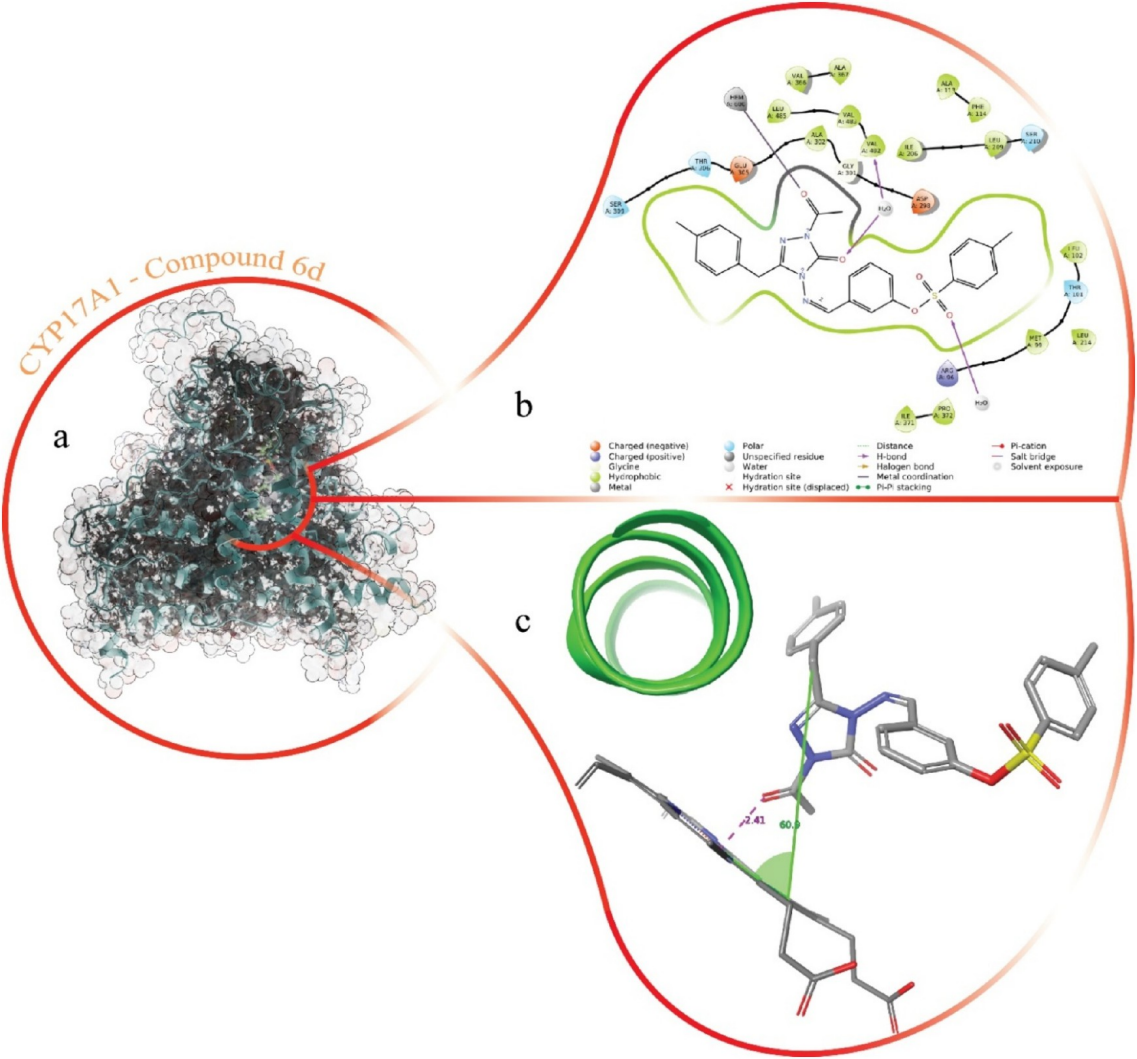
Images of CYP17A1-**6d** complexes formed by molecular docking study. (a) Compound **6d** binding site in the overall structure of CYP17A1. The outer surface of the protein structure has been rendered transparent as the binding site is completely buried. (b) 2D interaction map of the complex. (c) Angles and distances with a zoomed 3D view of the interaction with the HEM group.

The molecular docking analysis demonstrated that **6d** effectively establishes coordination with a Zn^2+^ ion, which is widely recognized as a key mechanism for inhibiting CA enzymes. This coordination was observed in both CAI and CAII isoforms, where the sulpho group of **6d** interacts directly with the Zn^2+^ ion. As depicted in Figure 4, within the active site of CAI, **6d** forms several stabilizing hydrophobic interactions with key residues. Specifically, the coordination with Zn^2+^ is facilitated by the sulpho group’s oxygen atoms, while the aromatic ring and adjacent methyl group align favorably with the hydrophobic environment created by residues Leu141, Ala142, Val143, He119, and Ala121. Additionally, hydrogen bonding interactions were identified between **6d** and residues Thr199 and Trp123. The carbonyl oxygen of the ketone group extending from the triazole ring fits well into a pocket near Trp123, forming a stable hydrogen bond. Furthermore, the region of **6d** containing the imine group is anchored through a π–π stacking interaction with His200, extending toward the sulpho group. This interaction stabilizes the orientation of the imine-containing region, ensuring a precise fit within the enzyme’s active site. As a result, **6d** is firmly positioned, supported by a combination of hydrophobic, hydrogen bonding, and π–π interactions, which likely contribute to its inhibitory potency.

**Figure 4:**
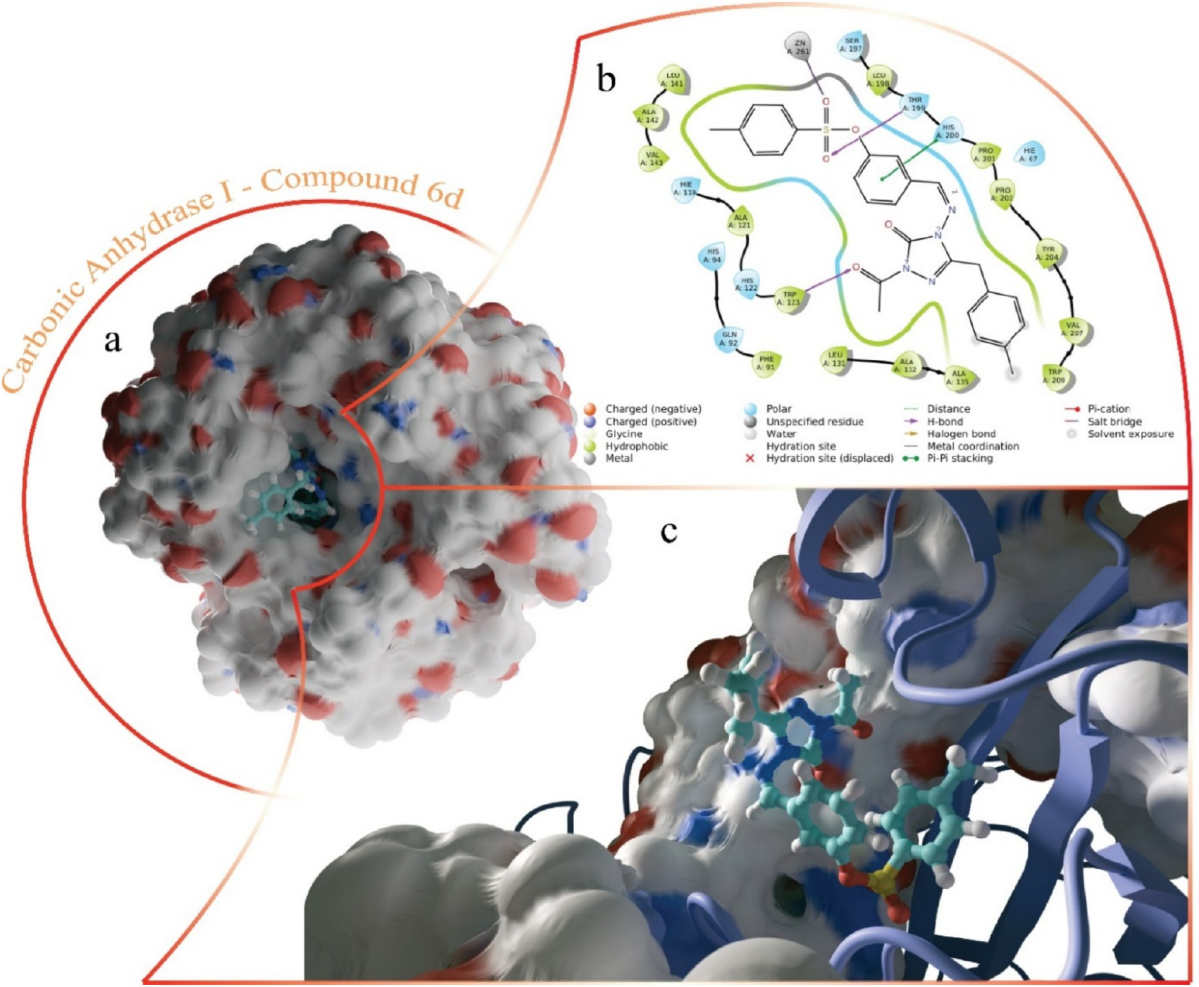
Images of CAI-**6d** complexes formed by molecular docking study. (a) Compound **6d** binding site in the overall structure of CAI. (b) 2D interaction map of the complex. (c) The 3D structure of the binding site is shown, ignoring part of the protein structure.

In the CAII-**6d** complex, a portion of **6d** extends beyond the protein structure, interacting with the surrounding solvent, as depicted in Figure 5. This positioning allows the compound to remain partially exposed to the external environment while still engaging with the active site. In addition to coordinating with the Zn^2+^ ion via the sulpho group’s oxygen atoms, another oxygen atom in the sulpho group forms hydrogen bonds with polar residues Thr199 and Thr200. Furthermore, the carbonyl groups near the triazole ring exhibit a strong tendency to establish hydrogen bonds with adjacent residues. These interactions are mediated by water molecules situated at the entrance of the active site, which facilitate bonding and stabilize the compound’s positioning within the enzyme.

**Figure 5:**
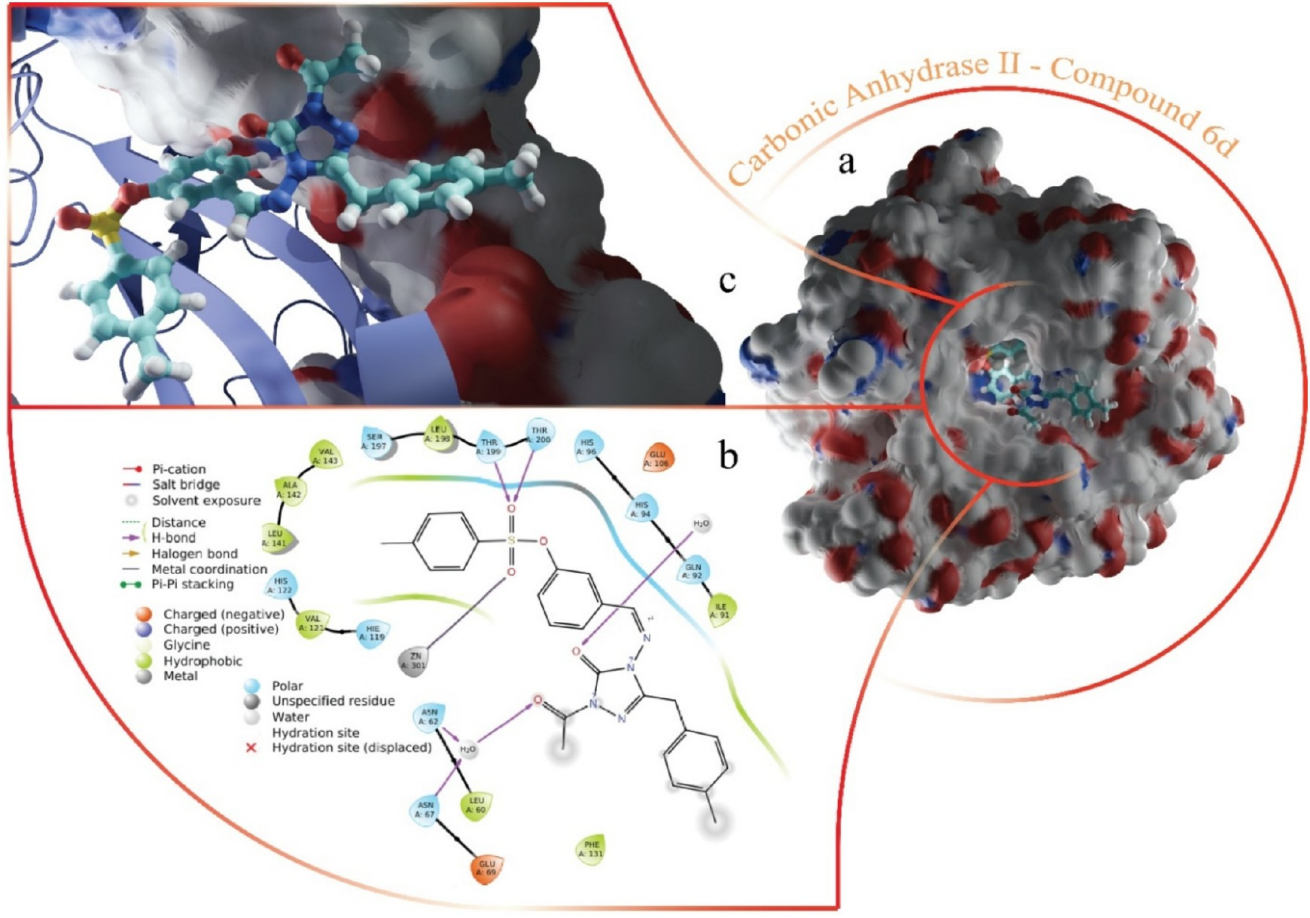
Images of CAII-**6d** complexes formed by molecular docking study. (a) Compound **6d** binding site in the overall structure of CAII. (b) 2D interaction map of the complex. (c) 3D structure of the binding site is shown ignoring part of the protein structure.

The results from the QM/MM molecular docking analysis revealed that **6d** demonstrates a preference for specific conformations. When **6d** adopts the “Z” configuration near the imine group, it allows the carbonyl moiety within the triazole ring to easily align with the HEM region of the CYP17A1 protein. This close proximity and specific angular orientation relative to the HEM group—a hallmark of effective CYP17A1 inhibitors as documented in previous studies—suggest that **6d** exhibits the necessary structural properties to act as an inhibitor. Interestingly, curcumin, a known CYP17A1 inhibitor from natural compounds previously studied in the PDB ID:3RUK model, showed notable structural similarities to **6d**.^102^ Curcumin’s phenolic oxygen atom, positioned 2.4 Å from the HEM iron, resembles steroid substrates.^102^ In synthetic chemotypes, it is well-documented that the nitrogen atom typically forms an angle of 60 degrees relative to the HEM plane.^102,103^ In **6d**, the aromatic ring branching from the triazole group toward the protein’s helical structure, along with the methyl group extension, achieves a comparable angular alignment of 60.9 degrees to the HEM plane. This structural data supports the hypothesis that **6d** could serve as a hybrid chemotype, integrating features of both natural CYP17A1 inhibitors and steroidal inhibitors.^102–104^ The calculated spatial orientation and angular positioning relative to the HEM group align well with the design strategy, suggesting that **6d** effectively combines inhibitory characteristics from both classes.

Regarding its interaction with carbonic anhydrase enzymes, the docking analysis further explains the *in vitro* activity observed for **6d**. The coordination of Zn^2+^ with oxygen atoms in the sulfo groups parallels the interactions seen with nitrogen atoms in sulfonamide-based inhibitors of carbonic anhydrases. This suggests that the predicted complex formation in the docking study was accurate, as the activity data corroborate these molecular interactions in a consistent manner. Additionally, the docking study highlighted the interactions of **6d** with both hydrophilic and hydrophobic regions in the CAI enzyme. Notably, the Thr199 residue played a significant role in stabilizing these interactions, which is reminiscent of the binding behavior seen with acetazolamide, a known carbonic anhydrase inhibitor. In the case of **6d**, the aromatic and methyl groups effectively occupied the hydrophobic pocket of the active site. Similarly, analogous interactions were observed in the CAII enzyme due to the structural resemblance between the two carbonic anhydrase isoforms, supporting the notion that **6d** can act as a potent dual-target inhibitor.

The electronic structure of **6d** was found to undergo significant alterations upon forming complexes with the CYP17A1, CAI, and CAII proteins, primarily due to coordination with the HEM group and Zn^2+^ ion, respectively. Notable changes in electronic configuration were observed, as illustrated in Figure 6, where the shapes of the HOMO and LUMO were visualized. These configurations were derived from QM calculations performed using the Qsite module, along with Single Point Energy (SPE) calculations where the ligand conformation within the complex was held constant. The results indicated that the HOMO–LUMO energy gap decreased from 0.24742 eV in the protein-bound state to 0.17006 eV in the SPE calculation, reflecting significant changes in electronic properties upon binding.

**Figure 6:**
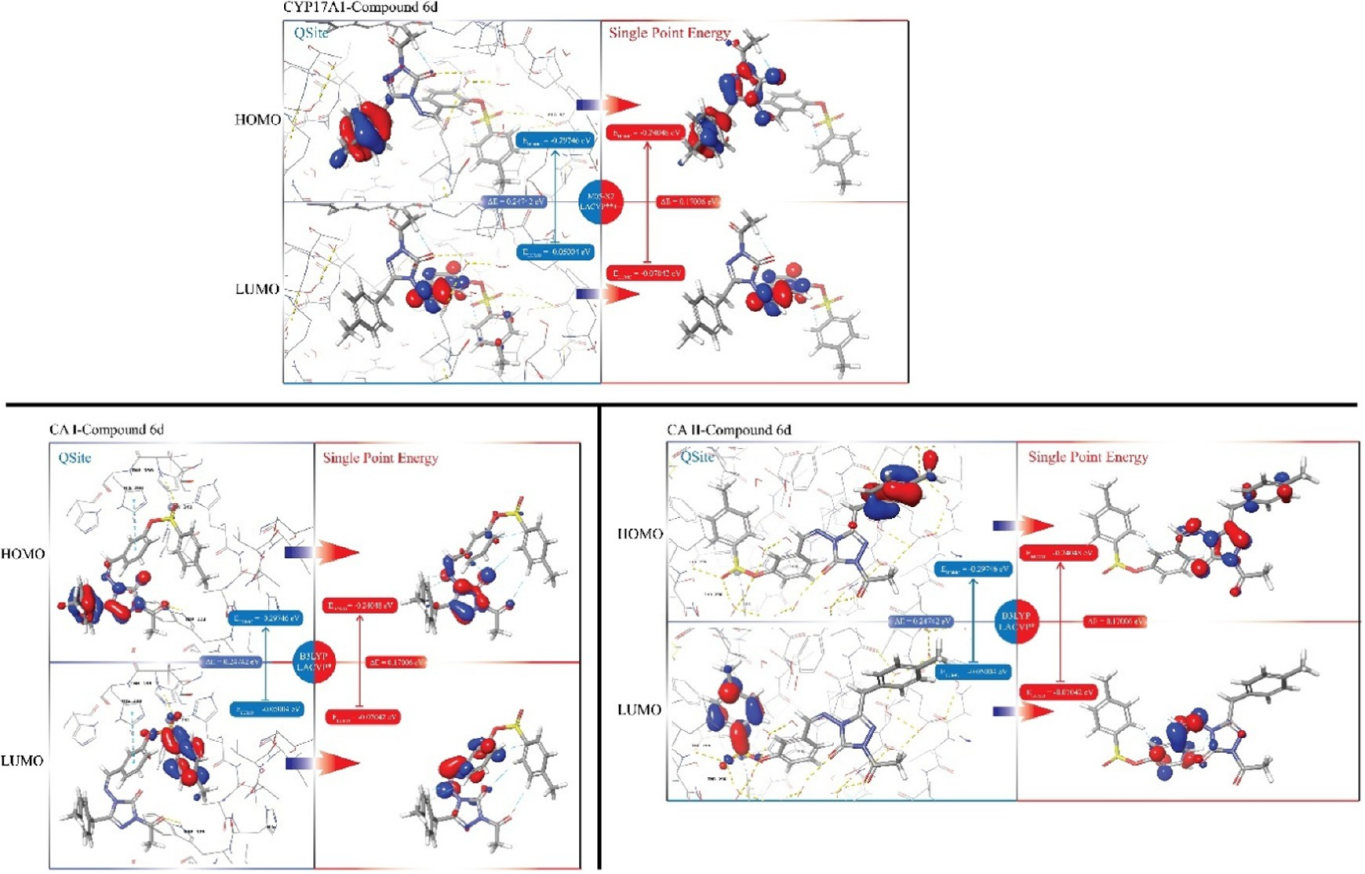
HOMO and LUMO orbital positions of ligands in protein–ligand complexes whose positions were subjected to final update by QM/MM docking.

In the CYP17A1-**6d** complex, the HOMO was localized on the aromatic ring and triazole group according to the SPE calculations, while the LUMO was consistently centered on the imine group in both Qsite and SPE evaluations. However, noticeable shifts in orbital shapes and sizes were observed between these two methods. For the CAI complex, Qsite calculations showed the HOMO concentrated on the aromatic ring and triazole. However, in the SPE calculations, the HOMO was no longer present on the aromatic ring; instead, the orbitals on the triazole underwent significant expansion and shape changes. The LUMO, initially located on the sulpho group and the attached aromatic ring in the Qsite analysis, shifted predominantly to the imine group in the SPE calculation. A similar pattern was observed in the CAII complex. The Qsite calculations placed the HOMO on the aromatic ring attached to the triazole. In contrast, the SPE calculation revealed that the HOMO was now distributed across the imine, triazole, and aromatic ring. Additionally, the LUMO, which was initially associated with the aromatic ring in the Qsite analysis, was found to shift toward the imine group in the SPE calculations.

The SPE analysis of the HOMO and LUMO orbitals, using Qsite, highlighted notable shifts in their distribution in the final conformations. These shifts indicate that the electron density concentrated on the 1,2,4-triazole ring is crucial for driving interactions throughout most of the molecule. Interestingly, the energy gap between the HOMO and LUMO orbitals is smaller when compared to that observed in the isolated protein structure and in single-point calculations. This smaller energy gap suggests that, within the protein environment, **6d** exhibits a more adaptable electron configuration. This increased electronic flexibility enhances the compound’s reactivity, allowing for stronger and more efficient interactions with the protein structure. These findings point to the importance of electron density modulation in optimizing the binding affinity of **6d**.

#### 3.4.2 Molecular Dynamics Study

MD simulations were performed for 100 ns to confirm the findings from the molecular docking studies and to analyze the stability of interactions between the protein–ligand complexes. Additionally, these simulations provided insights into the ongoing impact of **6d** on the structural integrity of the proteins. Trajectory analyses revealed that the initial binding positions of **6d** remained stable throughout the simulations for all protein–ligand complexes. The binding poses exhibited minimal deviations and fluctuations, indicating a strong and consistent interaction between **6d** and the respective protein targets.

##### 3.4.2.1 Molecular Dynamics Study for CYP17A1, CAI, and CAII

An MD simulation was performed on the CYP17A1 protein using two systems: one based on crystal structure data containing the abiraterone ligand and another involving the CYP17A1-**6d** complex refined via QM/MM docking. The simulation results indicated that the interactions near the HEM group were largely similar between the two complexes. However, a significant difference was observed in the CYP17A1-**6d** complex, which notably lacked interaction with the Asn202 residue, a contact present in the abiraterone-bound structure. Despite this discrepancy, **6d** displayed interaction patterns with surrounding residues near the HEM group that were comparable to those of abiraterone. Throughout the MD simulation, **6d** demonstrated rotational mobility, particularly in its sulpho group and attached aromatic ring, which rotated around its axis while remaining confined to a specific region within the protein structure. The N–N bond between the triazole and imine groups exhibited slight rotational flexibility. In contrast, the aromatic ring linking the imine and sulpho groups remained largely stationary due to a strong π–π interaction with the Phe114 residue. Similarly, the aromatic ring extending from the triazole to the benzyl group was constrained within a narrow binding pocket, maintaining a stable orientation. Additionally, the two segments of the CYP17A1 protein that extend into the membrane appeared to influence the overall dynamics and flexibility of both complexes. Despite these interactions, the protein structure displayed general stability, resisting major conformational changes throughout the simulation. Figure 7 illustrates the interaction of CYP17A1 with the membrane as well as the movement and conformational dynamics of **6d** within the binding site.

**Figure 7:**
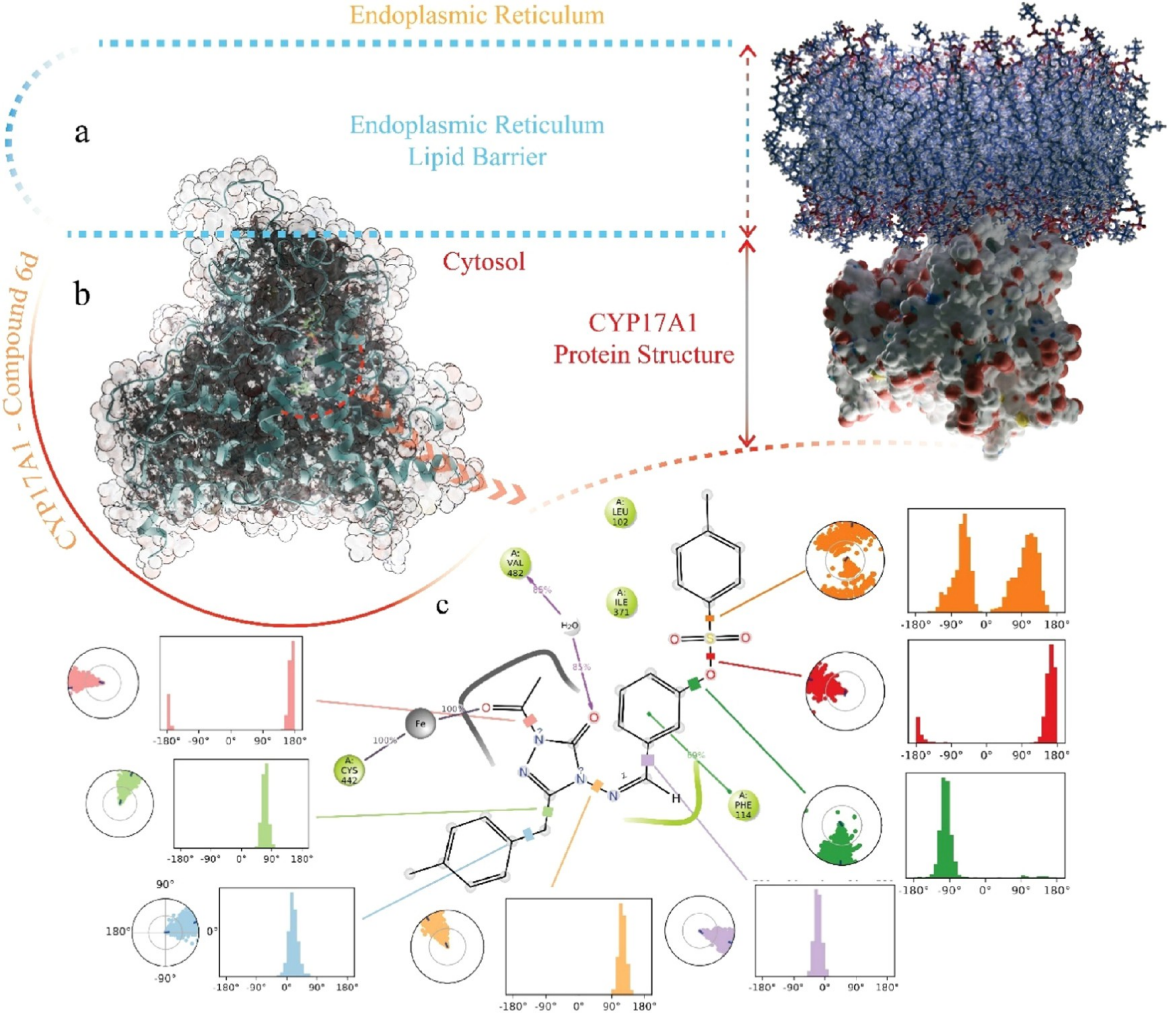
Structural and interaction analysis of **6d** with the human CYP17A1 enzyme within the endoplasmic reticulum membrane environment. (a) Schematic representation of the endoplasmic reticulum (ER) lipid bilayer embedding CYP17A1. (b) 3D structure of the CYP17A1 protein illustrating the binding pocket occupied by compound **6d** (highlighted in red), embedded in the ER membrane and visualized through a surface and ribbon model. (c) 2D interaction map of compound **6d** in the active site of CYP17A1, showing key residues involved in hydrophobic contacts, hydrogen bonding, and π–π interactions. Accompanying circular plots and histograms represent the dihedral angle distributions of ligand torsions during molecular dynamics simulations, indicating conformational stability and preferred binding orientations.

Figure 7 is given that the scale shows the membrane area to which CYP17A1 is bound throughout the MD simulation. The position and structure of the CYP17A1 protein are shown by ribbon structures with the protein surface area made transparent (Figure 7b). Red dashed lines indicate the exact position of **6d**. The residues and atoms that **6d** interacts with for more than 20% of the 100 ns MD simulation are shown along with the conformation change of the compound throughout the simulation (Figure 7c).

Trajectory analysis of the 100 ns MD simulations for the CAI and CAII complexes with **6d** indicated that the regions where the ligand was deeply embedded underwent minimal conformational changes. Compound **6d**, which adopted a similar planar conformation in both protein structures and maintained coordination through the same functional group, displayed increased solvent exposure in the CAII complex. This greater solvent interaction was particularly noticeable in regions of the ligand extending beyond the protein surface. In the CAII complex, this interaction with the surrounding solvent enhanced the mobility of **6d** within the active site, thereby increasing the flexibility of the binding cleft. A similar but less pronounced effect was observed in the CAI complex, where the smaller solvent-exposed area limited the impact on the ligand’s movement. Overall, the increased solvent interactions contributed to significant flexibility and dynamic behavior of **6d** within its binding sites. Notably, **6d** demonstrated interactions comparable to those of the reference inhibitor, acetazolamide, in both CAI and CAII complexes. However, it occupied a larger volume within the active sites, maintaining stable interactions throughout the simulation. This expanded interaction footprint allowed **6d** to establish a more extensive and sustained engagement with the protein structures compared to acetazolamide.

##### 3.4.2.2 Root Mean Square Deviation (RMSD)

To investigate the key conformational changes in protein structures throughout the MD simulations, root-mean-square deviation (RMSD) analysis was performed. This approach compares the average positions of the protein structures in the initial simulation frames with their average positions in subsequent frames, quantifying positional deviations over time.^71^ To gain deeper insights into the conformational dynamics of CAI and CAII in the presence of the reference ligand, an autocorrelation analysis was applied to the RMSD data obtained from the simulations.^105^ This method allowed for a more detailed understanding of the consistency and patterns in the protein’s structural movements. In addition, instead of relying on traditional Fast Fourier Transform (FFT) techniques, power spectral density (PSD) estimation was conducted using the Welch method to analyze the RMSD data.^105,106^ This approach was chosen to reduce variance and enhance the accuracy of frequency-domain analysis. A detailed representation of these analyses, including the RMSD, autocorrelation, and PSD results, is provided in Figure 8.

**Figure 8:**
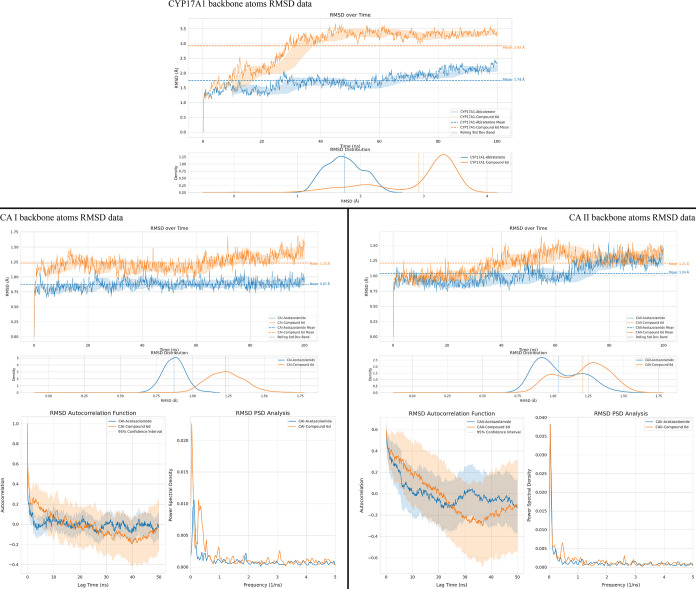
RMSD data of protein backbone atoms in MD simulations of complexes formed by CYP17A1, CAI, and CAII structures with reference ligands and **6d**.

The RMSD analysis for the CYP17A1 protein structure showed stabilization after 30 ns of simulation. The CYP17A1-**6d** complex exhibited an initial RMSD of approximately 2 Å, reaching 3.5 Å by the end of the simulation, with an average RMSD of 2.92 Å. The standard deviation range for the **6d** complex narrowed toward the end, indicating reduced fluctuations compared to earlier stages. Additionally, the RMSD distribution graph for the **6d** complex showed a tighter distribution, with higher frequency occurrences of specific RMSD values compared to the abiraterone-bound complex. When comparing the RMSD values of backbone atoms for carbonic anhydrase enzymes, it was observed that complexes with **6d** demonstrated RMSD profiles similar to those formed with the reference inhibitor, acetazolamide, particularly in the CAII enzyme. For CAI, the mean RMSD for the **6d** complex was 1.23 Å, while for the acetazolamide complex, it was 0.87 Å. In the CAII enzyme, the mean RMSD was 1.21 Å for **6d** and 1.04 Å for acetazolamide. Notably, the standard deviation bands remained stable, suggesting that both enzymes maintained a degree of structural flexibility.

The RMSD scatter plots indicated that the acetazolamide complex in CAI exhibited a narrower distribution range, while both **6d** and acetazolamide complexes in CAII showed similar effects at different simulation time intervals. Autocorrelation analysis revealed that acetazolamide complexes rapidly approached zero, indicating minimal periodic behavior. In contrast, the **6d** complexes maintained negative autocorrelation values over time, suggesting periodic oscillations in protein movements. The PSD analysis, which breaks down the RMSD signals into frequency components, showed that the **6d** complexes exhibited higher frequency components. This finding indicates faster and more extensive structural adjustments, reflecting increased flexibility and dynamic behavior within the protein–ligand complexes.

##### 3.4.2.3 Root Mean Square Fluctuation (RMSF)

The RMSF plots, which quantify the local flexibility and regional stability of protein–ligand complexes, are detailed in Figure 9. These plots compare the MD simulation results for the complexes formed with reference compounds and **6d**. In the case of CYP17A1, the RMSF values for both the reference and **6d** complexes were largely similar. However, **6d** was observed to reduce local fluctuations, particularly around residue Glu230 (residue index ∼ 240), where it produced a lower peak. For the CAI enzyme, **6d** led to increased local fluctuations in several regions, suggesting greater flexibility induced by the ligand. Meanwhile, in the CAII complexes, the protein structures displayed multiple prominent peaks, indicating enhanced localized movement in response to ligand binding.

**Figure 9:**
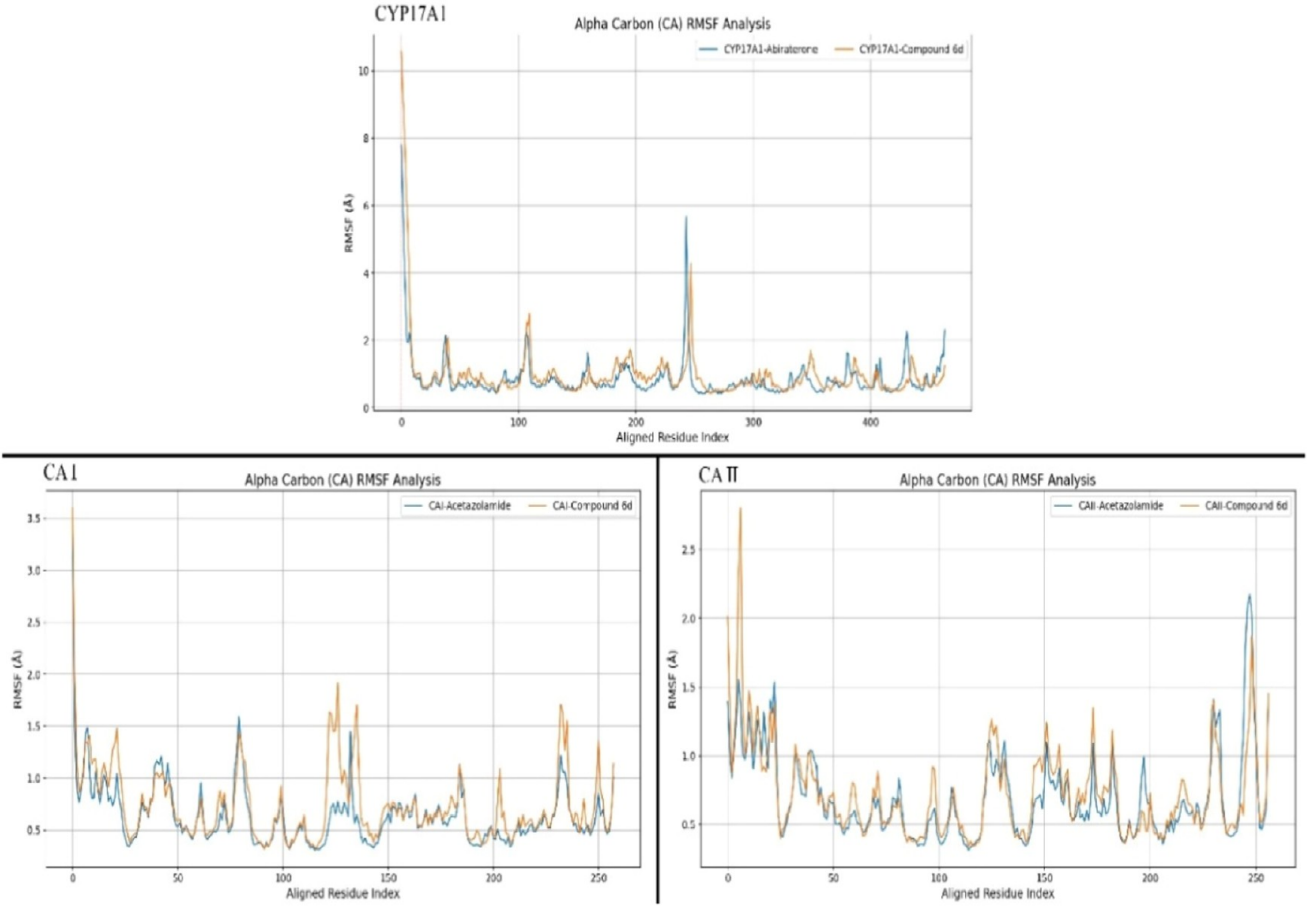
RMSF plots of protein α carbons of reference ligands and **6d** for each enzyme used in the MD study.

MD simulations conducted with the protein–ligand complexes revealed that **6d** maintained a stable and consistent dynamic behavior throughout the simulations. Notably, the CYP17A1 protein preserved its interactions with the membrane across the entire simulation time frame, aligning with established data reported in the literature. This stability confirms the reliability and accuracy of the constructed system.

In the simulations involving CAI and CA II enzymes, **6d** exhibited slight oscillatory movements within the active site in a solvent environment. This subtle motion triggered a cascading effect on the ligand’s positioning, particularly in the CAII-**6d** complex. The impact of this dynamic behavior was reflected in the RMSD data, where the increased variability in the deviation bands was more pronounced for complexes involving **6d**. This fluctuation suggests an enhancement in the flexibility of the binding site and its adjacent residues, thereby improving the interaction potential between **6d** and surrounding residues. Such flexibility is advantageous, as it helps maintain the substrate binding site for an extended duration, effectively supporting the inhibition process.

Moreover, the periodic fluctuations observed in the CYP17A1 protein structure’s RMSD data indicate a reduction in overall variability, despite reaching a higher RMSD value compared to the abiraterone complex. This plateauing behavior suggests that the CYP17A1-**6d** complex stabilizes into a distinct conformational state that, while different from that of abiraterone, exhibits greater structural stability.

The autocorrelation and PSD analyses indicate that both carbonic anhydrase enzymes exhibit periodic motion, as reflected in the autocorrelation graphs dropping below zero. However, the PSD data reveal that the complex with **6d** undergoes more rapid structural adjustments. These findings, together with the RMSD values, imply that the compound reaches a stable conformation relatively quickly. Clear interpretations were primarily possible for the CYP17A1-**6d** complex using the RMSF graphs. In contrast, the CAI and CAII complexes did not show fluctuation patterns comparable to those seen in reference compound complexes. In the case of CYP17A1 complexes, the introduction of **6d** produced a dampening effect around the amino acid index of 250, a region known for its sensitivity to structural fluctuations. This effect suggests that **6d** not only enhances binding affinity but also promotes additional occupancy of the active site and interaction with the HEM group, likely due to its conformational rigidity.

#### 3.4.3 Protein Dynamics

##### 3.4.3.1 MM-GBSA Supported Analysis of Protein Dynamics

To investigate protein dynamics, the MD simulation data were utilized to identify metastable states and to compare stable conformations with those induced by reference compounds. The MM-GBSA free energy values, calculated for each frame, were integrated into a diffusion map analysis. This method leverages the precise (*x*, *y*, *z*) atomic coordinates of each protein frame, enabling a detailed exploration of conformational changes throughout the MD simulations. Diffusion mapping, a dimensionality reduction technique, was applied to uncover fundamental transition pathways and collective motions while preserving the local geometric structure of the protein system. By constructing a similarity matrix based on molecular positions over time and performing eigenvalue decomposition, low-dimensional coordinates were derived, facilitating the visualization of transitions between distinct structural and dynamic states. Incorporating MM-GBSA-derived free energies into this method allowed for more realistic analyses, addressing limitations that may arise with purely probability density-based energy estimations. The clustering of conformations was performed using the OPTICS algorithm, which effectively minimized subjective bias in identifying stable states. Furthermore, additional analyses, including UMAP and TICA-FES, were conducted in line with established literature standards for probability density estimation.^107^ To capture conformational transitions in the protein structures, MSM were successfully employed, enabling a comprehensive understanding of dynamic behavior across different protein states.

The MD simulation data of the CYP17A1 protein structure in complex with abiraterone revealed the presence of four distinct basic conformations, as identified by the OPTICS algorithm. In the diffusion map, in which the energy calculations for these conformations were integrated, it was observed that the lowest energy values were concentrated in two conformations. The two basic conformations are illustrated in Figure 10 in pink and blue. The frame numbers indicate that these two conformations are realized consecutively. The remaining two conformations were identified as metastable states, as they exhibited higher energy frames. In the CYP17A1-**6d** complex, a single conformation exhibiting high stability dominated the simulation after 20 ns. As illustrated in Figure 10, the energy values of this conformation indicate that low-energy frames were collected after 18 ns. The high-energy frames appear to constitute an unstable transition state from the outset of the simulation until the 18th nanosecond. Upon independent analysis of the MM-GBSA plots in the diffusion map, it was observed that **6d** exhibited a markedly lower energy profile than abiraterone in the context of the CYP17A1 protein structure. MM-GBSA calculation results and docking scores are shown in Table 3. In addition, the hydrogen bonding interactions during the MD simulation are illustrated in detail for each complex in Figures S19–S21.

**Figure 10:**
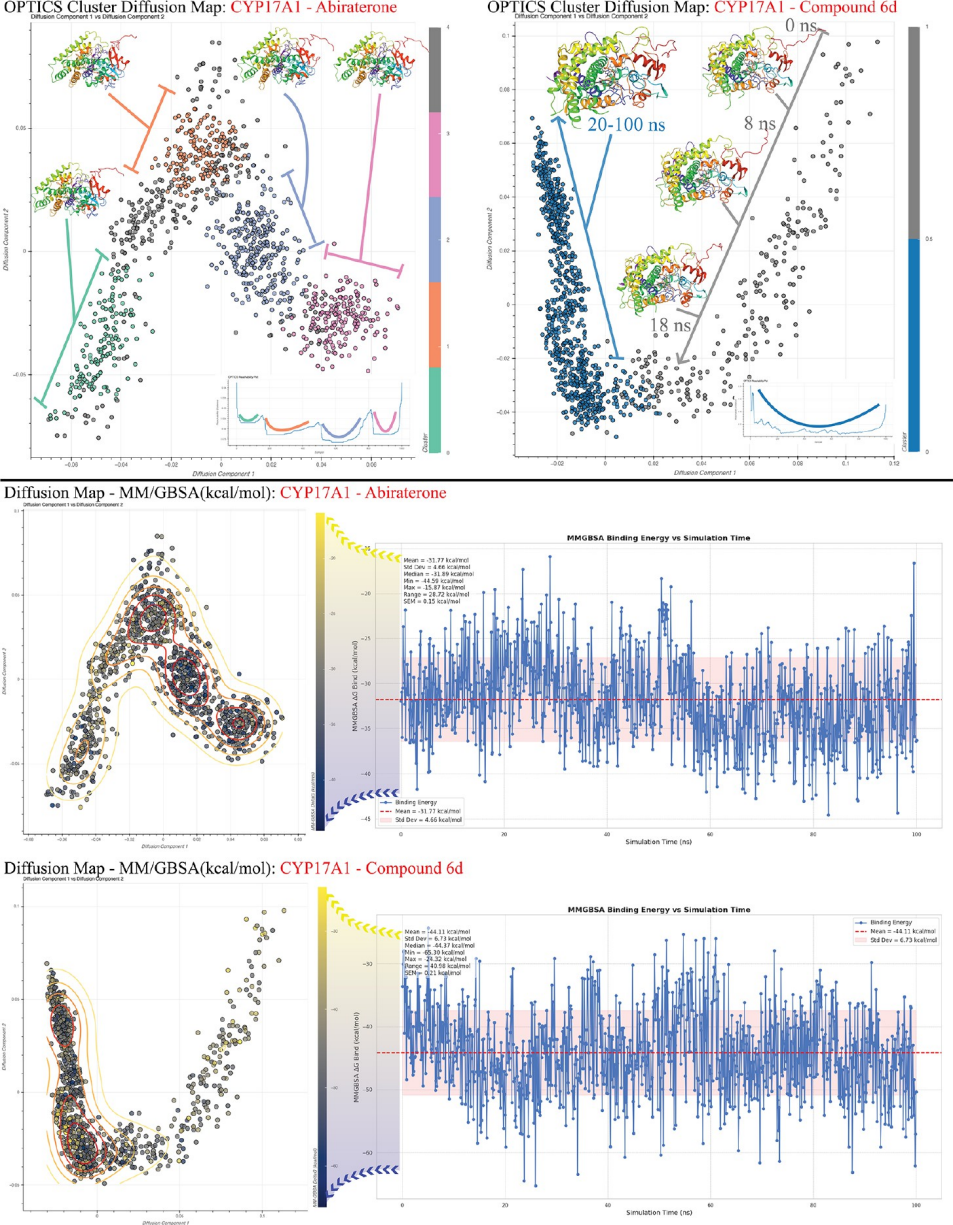
Graphs showing the conformation space of the complexes formed by CYP17A1 with **6d** and abiraterone compounds with OPTICS clustering algorithm and MM-GBSA graphs and diffusion maps containing energy values integrated with these graph data.

**Table 3:** Molecular Docking Scores and MM-GBSA Calculation Results (in kcal/mol)

compound	docking score			MM-GBSA free energy mean		
	**CA I**	**CA II**	**CYP17A1**	**CA I**	**CA II**	**CYP17A1**
**6d**	–9.196	–9.605	–9.798	–36.90	–49.09	–31.77

Based on preliminary analyses of MD simulations, it can be inferred that **6d** demonstrates superior inhibitory efficacy compared to the reference compounds. However, while the general mechanism of inhibition has been elucidated, the precise impact of **6d** on fine-tuning protein dynamics within this mechanism remains less understood. Given the critical importance of these nuances for drug development and understanding pharmacological effects, a deeper analysis of protein dynamics was pursued. To achieve this, conformational transitions were analyzed using the diffusion map method, known for its sensitivity, coupled with energy evaluations from MM-GBSA calculations. This approach proved particularly insightful for CYP17A1 complexes. Notably, **6d** was observed to achieve a stable conformation relatively quickly, within about 20 ns. This rapid stabilization suggests that the protein’s conformational space becomes more restricted upon binding, allowing the ligand to remain securely in the active site for an extended period. In contrast, the reference compounds exhibited more frequent conformational shifts, potentially leading to the ligand drifting away from the active site.

The diffusion map analysis revealed distinct clustering patterns in the CYP17A1-abiraterone complex, indicating the existence of two accessible conformations with transitions between them. Conversely, the CYP17A1-**6d** complex formed a single, tightly packed cluster, suggesting a stable conformational state. Protein structures sampled from this cluster showed consistent similarity across all frames, even in flexible regions, indicating that a stable conformation is maintained throughout the simulation. This stability is reflected in the RMSD data, which plateaued after approximately 20 ns, indicating that the protein–ligand complex had settled into a stable configuration. In contrast, the CYP17A1-abiraterone complex displayed an increasing RMSD trend, signaling the presence of multiple, less stable conformational states. MM-GBSA energy comparisons between the two complexes further confirmed that the **6d** complex is more stable overall.

The conformation space of the CAI enzyme was determined using both diffusion maps and the UMAP reduction method, as illustrated in Figure 11. In the UMAP analysis of the complex formed by **6d** with CAI, a single low-energy domain was observed, whereas in the UMAP analysis of the complex formed by acetazolamide, this low-energy domain was seen to spread and form several different stable conformations. The analysis with diffusion maps corroborates this conclusion and indicates the presence of a single basic conformation, depicted in orange in the OPTICS algorithm clustering analysis presented in Figure 11. The MM-GBSA integrated diffusion map indicates that the conformation depicted in orange exhibits the lowest energy frames. The remaining two conformations exhibit elevated energy fields, suggesting that the transition to these states may become increasingly challenging over time. The MM-GBSA plot allowed the CAI-**6d** complex to be determined to be stable with a low energy perspective exhibited at all times of the simulation.

**Figure 11:**
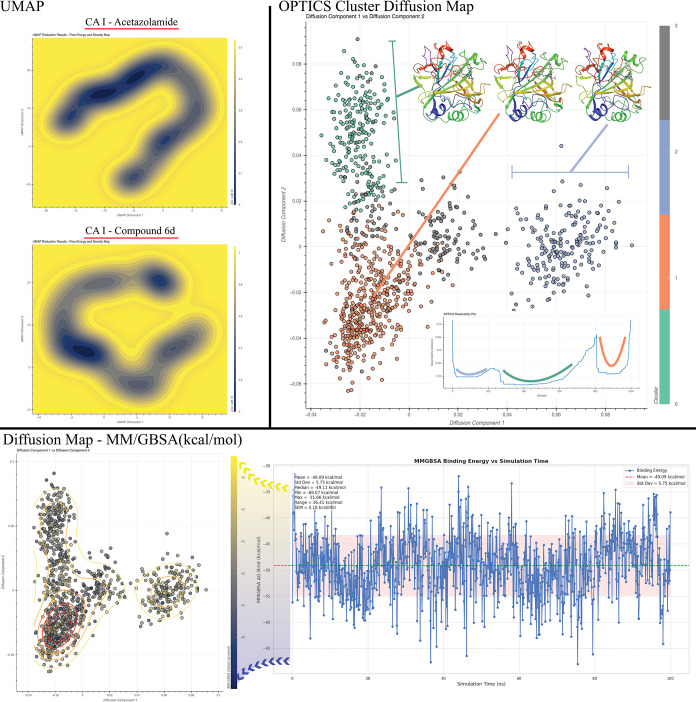
Comparative demonstration of UMAP reduction of CAI enzyme complexes formed by **6d** and acetazolamide compounds obtained from MD simulation data. Also, diffusion maps with OPTICS clustering algorithm and integration of data from the MM-GBSA graph.

The UMAP analysis of the CAI protein structure highlights that, while the complexes share some similarities, there are also distinct differences. Notably, the acetazolamide complex undergoes two prominent conformational transitions, characterized by deep energy wells that are closely intertwined. In contrast, the transitions observed in the **6d** complex are separated by significant energy barriers, indicating more restricted conformational changes. Further evidence from the OPTICS algorithm-based diffusion map revealed three distinct conformational states, suggesting that the high energy barriers limit transitions between these states. This explains the isolated, deep energy valley observed in the UMAP plot. Additionally, the clustering of low-energy frames into a single group on the MM-GBSA-enhanced diffusion map implies that transitions to the other conformations are highly challenging, reinforcing the stability of one dominant state. Over time, it is likely that the protein structure will settle into this stable conformation while maintaining its inherent flexibility.

As illustrated in Figure 12, the acetazolamide compound exhibits three primary conformations with a high energy barrier when interacting with CAII. From these conformations, it is evident that the compound forms two distinct, highly similar microconformations, as evidenced by the dark areas in the UMAP graph. With regard to CAII-**6d**, an examination of the diffusion maps and UMAP graph reveals the presence of only two analogous conformations. The extensive area between these conformations, which represents metastable states and is identified as noise by the OPTICS algorithm, suggests the existence of a single metastable state, as it signifies high-energy regions in the MM-GBSA integrated graph. This area is separated by high-energy barriers that are analogous to those observed in the Acetazolamide complex. It is evident that the acetazolamide complex exhibits three fundamental conformations, while the **6d** complex displays two predominant conformations and a metastable state. An evaluation of the data indicates that **6d** exhibits a greater degree of conformational constraint in comparison to acetazolamide. The MM-GBSA plot demonstrates that the CAII-**6d** complex is consistently identified as a low-energy complex throughout the simulation. The observed increase in energy value around 80 ns of the simulation is consistent with the areas identified as noise in the OPTICS algorithm. The data demonstrate that physical forces exerted on protein dynamics enable the potential transition to a metastable state. However, the structure that does not remain in the metastable state is partially constrained by conformational constraints and subsequently transitions to low-energy conformations. In the acetazolamide complex, this resulted in the metastable state being incorporated into protein dynamics as a basic conformation, thereby leading to the conclusion that its effect on protein dynamics is more limited.

**Figure 12:**
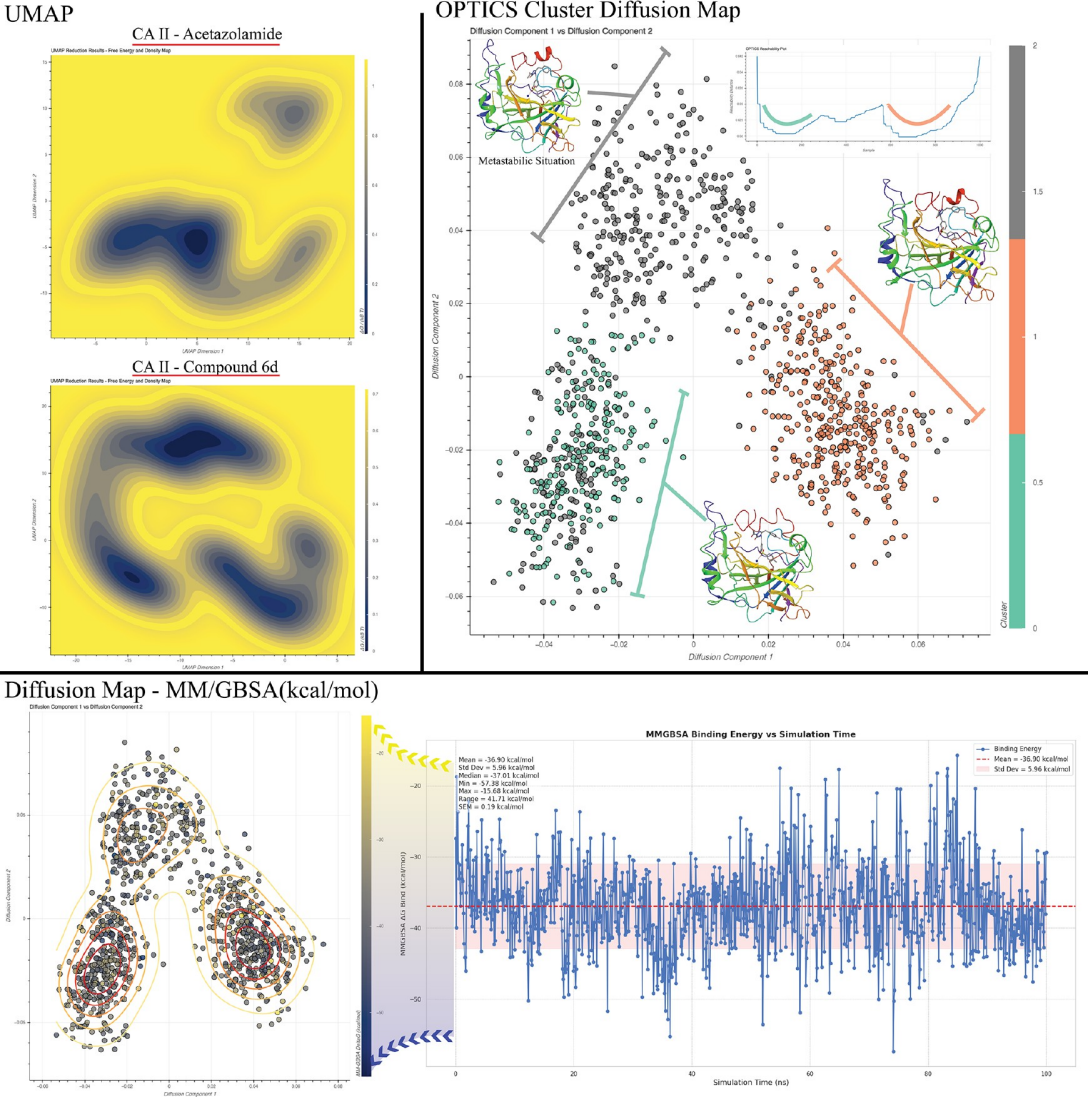
Comparative demonstration of UMAP reduction of CAII enzyme complexes formed by **6d** and acetazolamide compounds obtained from MD simulation data. Also, diffusion maps with the OPTICS clustering algorithm and integration of data from the MM-GBSA graph.

The MD simulations of the CAII enzyme revealed that the UMAP graph for the acetazolamide complex uniquely displayed a conformation surrounded by high-energy barriers. Although both the acetazolamide and **6d** complexes showed similarities within their low-energy valleys, the regions identified as noise by the OPTICS algorithm in the diffusion map analysis corresponded directly to the high-energy barriers observed in the UMAP graph. Interestingly, while the MM-GBSA energy calculations indicated a uniform distribution of frames, suggesting no significant conformational constraints, the probability density analysis favored two primary conformations. This apparent paradox can be attributed to the presence of deep energy valleys in the **6d** complex, which contributes to its stability.

#### 3.4.4 Markov State Models (MSM)

To investigate the long-term dynamics of the protein–ligand complexes obtained from molecular dynamics simulations, MSM were employed. This approach allowed for the development of a model that maps conformational transitions and quantifies the rates of these transitions, providing insights into the functional behavior of the protein structures. Implied time scale plots were constructed using hierarchical clustering, dividing the simulation data into 15 clusters and analyzing different lag times to capture the dynamics of state transitions. Additionally, TICA-FES analysis was performed with a lag time of 500 frames to assess the influence of ligands on extended processes like protein folding, focusing on three key components. The results of these analyses, which reveal the impact of ligand interactions on protein conformational dynamics, are illustrated for each complex in Figure 13.

**Figure 13:**
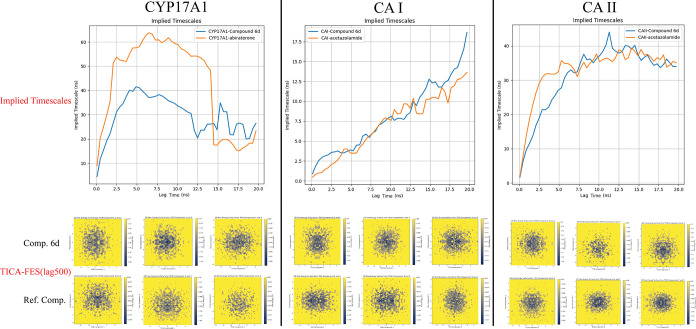
MSM and TICA-FES comparative images of MD simulated **6d** and reference compound complexes.

The CYP17A1-**6d** complex exhibited a rapid conformational shift early in the simulation, after which it stabilized into a dominant conformational state. This transition suggests a swift adjustment followed by steady-state dynamics. In contrast, the CYP17A1-abiraterone complex showed a gradual increase in its implied time scale plot, indicating more gradual changes in system dynamics. While both complexes displayed similar energy landscapes in the TICA-FES analysis, the energy profile defined by components 1 and 3 revealed deeper energy valleys for **6d**, suggesting a more stable state. The MSM analysis of conformational transition time scales revealed that while **6d** undergoes rapid transitions, it ultimately converges into a stable state. Additionally, the TICA-FES graphs for the **6d** complex displayed a greater number of deep energy wells, indicating its potential for sustained stability over longer time scales. This stability likely contributes to the inhibition of substrate binding and processing by ensuring the active site and HEM group remain occupied, thus preventing access for competing substrates. Overall, these findings highlight the robust inhibitory potential of **6d**, with enhanced stability and long-term effects compared to the reference compound, abiraterone.

For the CAI-**6d** system, the implied time scale plot showed a pattern similar to that of the acetazolamide complex, indicating analogous dynamics up to a lag time of 12.5 ns. However, beyond this point, the **6d** complex displayed a steeper increase, reflecting more constrained dynamics compared to acetazolamide. The TICA-FES plots further support this observation, demonstrating that **6d** induces a more focused accumulation of deep energy wells over time, enhancing its influence on long-term conformational stability. The acetazolamide complex is more prone to frequent conformational transitions due to the nearly equivalent energy levels of its two states and the absence of barriers between them. This dynamic behavior is supported by MSM analysis, where the **6d** complex exhibited slower transitions, indicating a more stable binding interaction. Moreover, the TICA-FES analysis revealed deep energy wells surrounding the **6d** complex, reflecting its stable, long-lasting interactions. The narrower range of energy fluctuations in this analysis suggests that the **6d** complex is stabilized by persistent, low-energy conformations, further emphasizing its potential for sustained inhibitory effects.

In the case of CAII, the implied time scale plots for both the **6d** and acetazolamide complexes indicated similar rates of change in system dynamics. However, the TICA-FES analysis revealed that acetazolamide produced deeper energy valleys at a lag value of 500 frames, suggesting a stronger impact on slow conformational motions, thereby contributing to a more stable dynamic state. Moreover, MSM analysis of conformational transition rates showed that both complexes exhibit comparable dynamics. However, the data suggest that the acetazolamide complex can traverse the high-energy barriers seen in the UMAP graph more readily. In contrast, the **6d** complex leverages the flexibility of the binding site as a strategic factor in its inhibitory mechanism, leading to fewer conformational transitions within the same time frame. This implies that **6d** stabilizes within the binding site more effectively, enhancing its inhibitory potency through reduced conformational shifts.

Based on the experimental results and computational analyses conducted in this study, our series of compounds, especially **6d**, appears to have promising therapeutic potential for the treatment of advanced prostate cancer, demonstrating high selectivity. The strong inhibitory activity against CAI and CAII enzymes may contribute to regulating pH levels within the tumor microenvironment, which is crucial for cancer progression. Moreover, there is a possibility of achieving a synergistic therapeutic effect if the CYP17A1 enzyme, which influences both cancer cell growth and “spontaneous androgen production”, becomes upregulated. However, fully exploring this synergistic potential requires further validation through *in vivo* studies. Therefore, our research group will next focus on conducting *in vivo* experiments to assess these effects in a biological context.

## 4 Conclusions

This study focused on the successful synthesis and thorough characterization of a new series of *N-*acetyl Schiff bases (**6a**–**e**), designed to develop compounds with potential therapeutic applications, particularly in prostate cancer treatment and human carbonic anhydrase isoforms I and II inhibition. The synthesis was efficiently carried out using previously synthesized Schiff bases (**5a**–**e**), and the structures of the new derivatives were confirmed through a combination of advanced spectroscopic techniques, including IR, ^1^H NMR, and ^13^C NMR spectroscopy, along with elemental analysis. The presence of characteristic vibrational bands and proton signals provided strong evidence for the formation of the targeted compounds, validating their successful synthesis with yields ranging between 65.71 and 85.71%.

The biological evaluation of these compounds demonstrated their selective cytotoxic effects against DU145 while exhibiting reduced toxicity toward PNT1a. Specifically, compounds **6a**, **6d**, and **6e** were found to significantly inhibit cancer cell viability in a dose-dependent manner, while exerting minimal impact on normal cells, as evidenced by their substantially higher IC_50_ values for PNT1a cells. This selective cytotoxicity suggests these compounds may act as promising antiprostate cancer agents with a potentially favorable safety profile. The selective targeting observed in cancer cells could be particularly advantageous in reducing side effects typically associated with conventional chemotherapy.

Furthermore, the newly synthesized compounds were evaluated for their inhibitory effects on hCAI and hCAII enzymes that are implicated in various physiological processes, including pH regulation and intraocular pressure control. The enzyme inhibition assays revealed that compounds **6a**, **6c**, and **6d**, in particular, exhibited potent inhibitory activity, with *K*_i_ values lower than that of the clinically used standard inhibitor, acetazolamide. Notably, compound **6d** displayed the most effective inhibition against both hCAI and hCAII, with IC_50_ values of 7.12 and 10.62 μM, respectively. This suggests that these compounds may have potential applications in the treatment of conditions like glaucoma, where reducing intraocular pressure is a key therapeutic goal.

To better understand the molecular interactions underlying the observed biological activities, an extensive series of computational analyses was performed, which included detailed QM/MM molecular docking studies. The docking studies revealed that **6d**, in particular, demonstrated a high binding affinity for the active sites of CYP17A1, hCAI, and hCAII enzymes. The compound established stable coordination with the Zn^2+^ ion in carbonic anhydrase isoforms and effectively interacted with key amino acid residues within the binding pockets. QM/MM studies further elucidated the binding modes, highlighting the strong coordination between the triazole moiety of **6d** and the active site Zn^2+^ions, as well as hydrophobic interactions that stabilize the ligand within the enzyme pockets.

The MD simulations over 100 ns confirmed the stability of the protein–ligand complexes, particularly for compound **6d**. The simulations showed that **6d** maintained its binding conformation with minimal fluctuations, indicating a stable interaction profile, especially within the active sites of the targeted enzymes. Additionally, the RMSD and RMSF analyses indicated that the presence of **6d** in the enzyme complexes contributed to the stabilization of key structural regions, reducing protein flexibility in a manner consistent with effective enzyme inhibition.

The molecular dynamics simulations, coupled with the MSM analysis, revealed that **6d** rapidly achieves stable binding conformations, particularly in the CYP17A1 enzyme. The reduced conformational flexibility and the deep energy wells observed in the TICA-FES and MM-GBSA analyses suggest that **6d** induces a conformational constraint that prevents substrate access, thereby enhancing its inhibitory potency. These findings are supported by the observed electronic reconfiguration upon ligand binding, which further stabilizes the compound within the enzyme’s active site. For the **6d**, MM-GBSA binding free energy values support the observed *in vitro* assays, showing relatively low and consistent values across the simulation timeline. Specifically, for CAI, the MM-GBSA values were −56.21 kcal/mol at the starting position and −38.93 kcal/mol at the final position; for CAII, −27.38 and −29.33 kcal/mol; and for CYP17A1, −33.58 and −50.33 kcal/mol, respectively. These values align with the *in vitro* IC_50_ data, which were 9.26  ±  0.88 μM for CAI, 11.72  ±  1.01 μM for CAII, and 49.80  ±  11.12 μM for CYP17A1. The close agreement between the MM-GBSA results and the biological activity supports the reliability of our computational approach.

This study highlights the therapeutic potential of newly synthesized *N*-acetyl Schiff bases, particularly **6d**, which demonstrates strong efficacy as a dual-target inhibitor with potential applications in prostate cancer treatment and inhibition of hCAI and hCAII enzymes. The combination of selective cytotoxicity toward cancer cells, robust enzyme inhibition, and stable binding interactions suggests that these compounds hold promise for further optimization as therapeutic agents. Our results indicate that **6d** may be beneficial in monotherapy approaches and/or in cases of drug resistance with the potential for a broad perspective multitargeted effect, while providing important evidence that it should be supported by further studies. Future research will prioritize *in vivo* studies to confirm their efficacy, safety, and pharmacokinetics, ultimately supporting their potential clinical use in cancer therapy and glaucoma management.

## Supplementary Material



## Data Availability

The data sets, scripts, and accompanying documentation utilized in this research are openly accessible at https://zenodo.org/records/14993673.

## Notes

^1^H and ^13^C NMR spectra of synthesized compounds **6a–e**; FT-IR spectra of compounds **6a–e**; molecular docking validation data for CAI (1AZM), CAII (3HS4), and CYP17A1 (3RUK); molecular dynamics (MD) interaction profiles of compound **6d** with CAI, CAII, and CYP17A1 (PDF)
