# Supplementary material for: Synthesis and Computational Evaluation of N‑Acetyl-Derived Schiff Bases Incorporating 1,2,4-Triazoles for Dual Inhibition of Prostate Cancer Cells and Carbonic Anhydrases
Source: ACS Omega. 2025 Aug 22;10(34):38585–608. doi: 10.1021/acsomega.5c03271 (PMC12409542; doi:10.1021/acsomega.5c03271)
Supplement: Supplementary file 1 [file ao5c03271_si_001.pdf]

Supporting Information for

Synthesis and Computational Evaluation of *N*-Acetyl-Derived Schiff Bases Incorporating 1,2,4-Triazoles for Dual Inhibition of Prostate Cancer Cells and Carbonic Anhydrases

Hilal Medetalibeyoğlu[a*], Abdulmelik Aytatlı[b,c], Sevda Manap[a], Abdurrahman Atalay[d*], Ahmet Buğra Ortaakarsu[e], Burak Tüzün[f*], Parham Taslimi[g], Özlem Gürsoy-Kol[h], Ömer Faruk Karataş[b,c], Haydar Yüksek[a]

[a]Department of Chemistry, Faculty of Arts and Sciences, Kafkas University, 36040 Kars, Turkey
[b]Molecular Biology and Genetics Department, Erzurum Technical University, 25100 Erzurum, Turkey
[c]High Technology Application and Research Center, Erzurum Technical University, 25100 Erzurum, Turkey
[d]Department of Nutrition and Dietetics, Faculty of Health Science, Avrasya University, 61080 Trabzon, Turkey
[e]Department of Chemistry, Faculty of Arts and Sciences, Gazi University, 06500 Ankara, Turkey
[f]Plant and Animal Production Department, Sivas Technical Sciences Vocational School, Sivas Cumhuriyet University, 58140 Sivas, Turkey
[g]Department of Biotechnology, Faculty of Science, Bartin University, 74100 Bartin, Turkey
[h]Department of Chemistry, Faculty of Science, Karadeniz Technical University, 61080 Trabzon, Turkey

Corresponding authors
*** Hilal Medetalibeyoğlu**
Department of Chemistry,
Faculty of Arts and Sciences,
Kafkas University, 36040 Kars,
E-mail: hilalmedet@gmail.com

*** Abdurrahman Atalay**
Department of Nutrition and Dietetics,
Faculty of Health Science,
Avrasya University, 61080 Trabzon,
E-mail: atalay.abdurrahman@yahoo.com

*** Burak Tüzün**
Plant and Animal Production Department,
Technical Sciences Vocational School of Sivas,
Sivas Cumhuriyet University, 58140 Sivas,
E-mail: theburaktuzun@yahoo.com.tr

Table of contents

^{1}H and ^{13}C NMR spectra of the synthesized compounds ………………………..............S2-S11.

IR spectra of the synthesized compounds ……………………………………………...…S12-S14.

Molecular docking validation results ………………………………………………....…S15-S16.

Molecular Dynamics (MD) simulation interaction profiles ….………………………...…S17-S19.

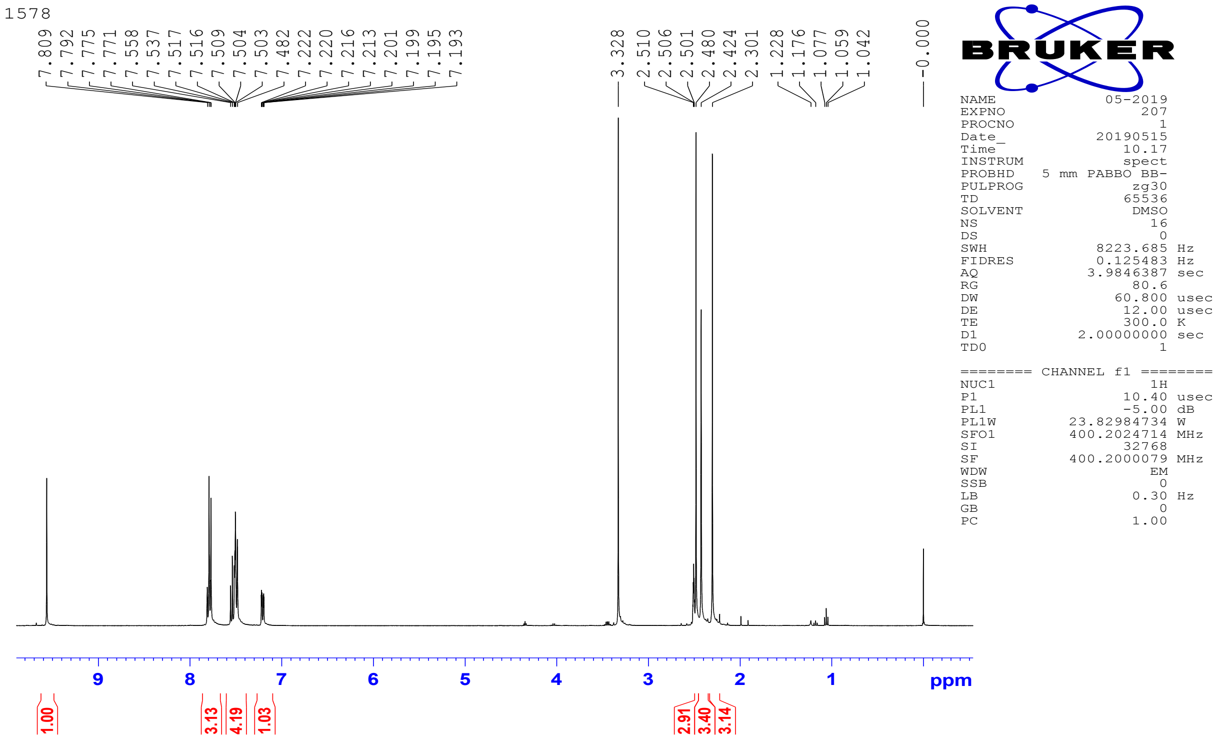

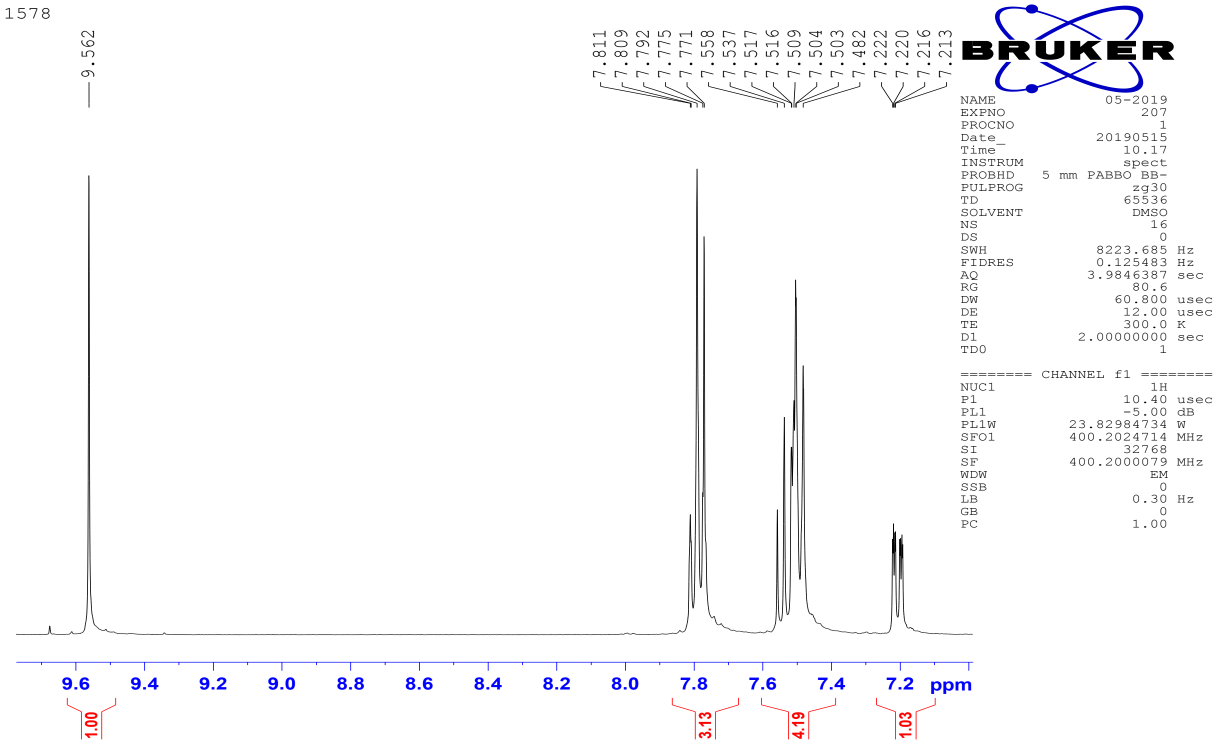

Figure S1. ^{1}H-NMR (400 MHz, DMSO) spectrum of compound **6a**.

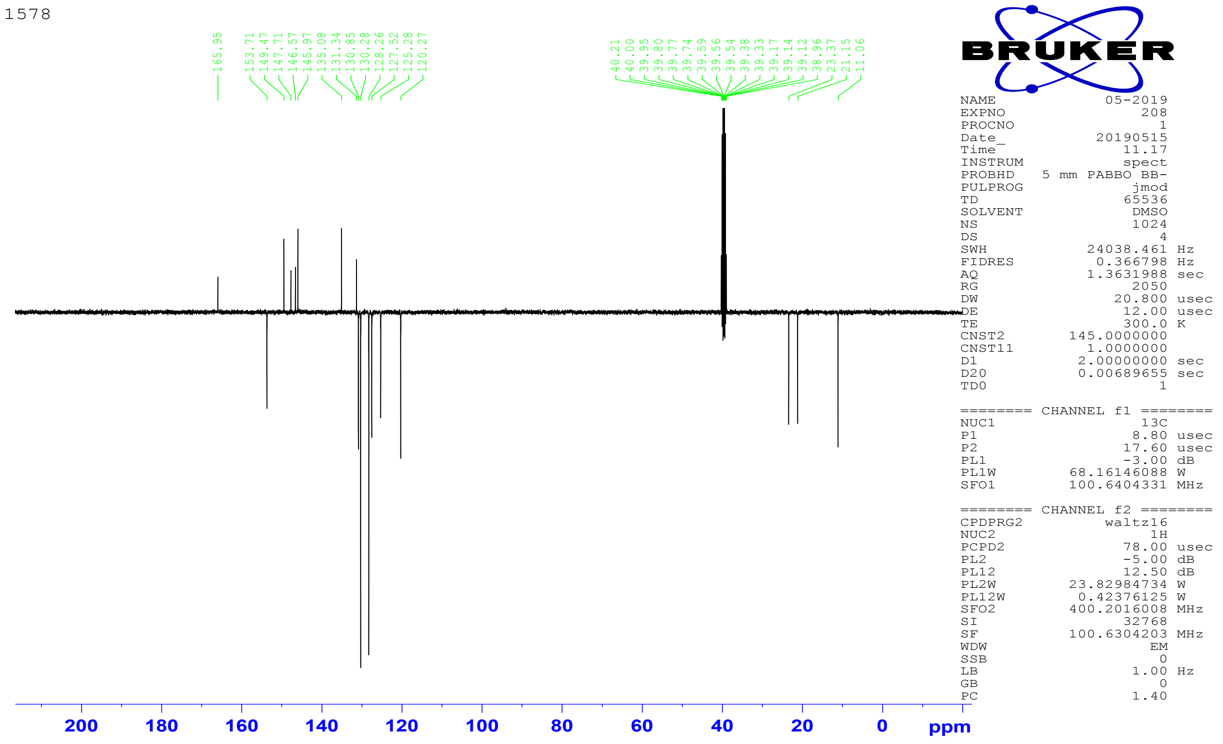

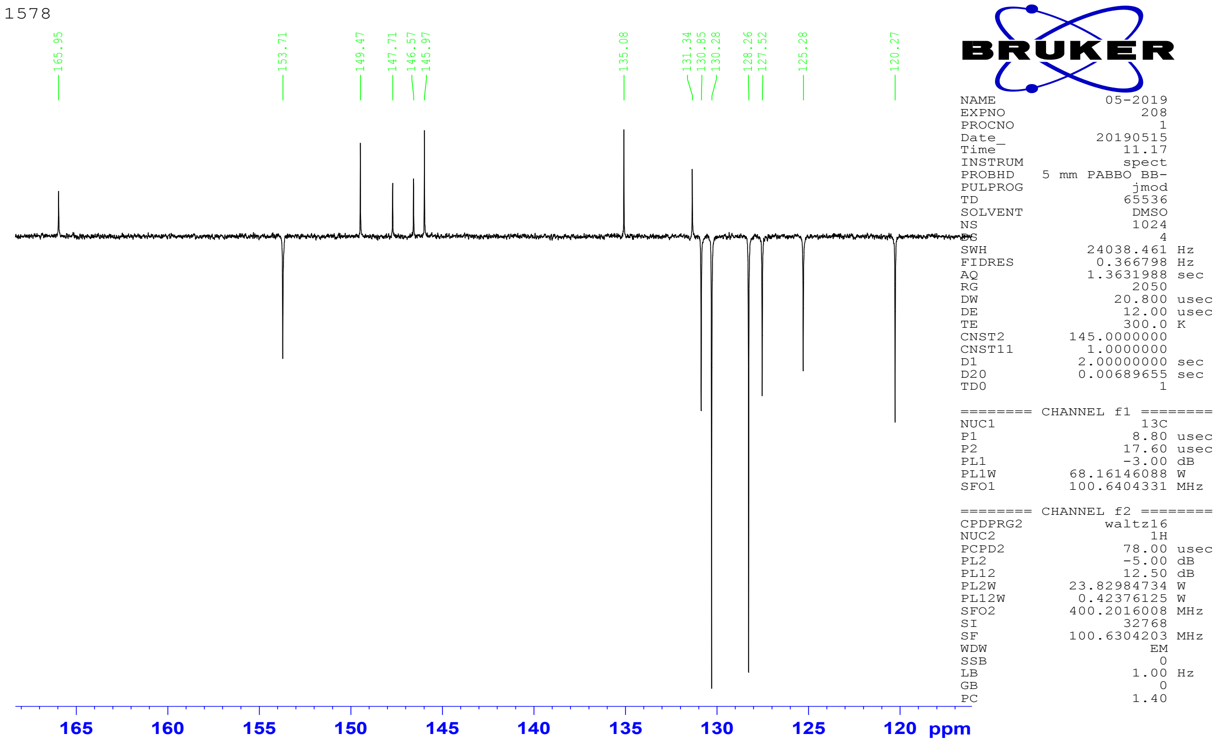

Figure S2. ^{13}C-NMR (100 MHz, DMSO) spectrum of compound **6a**.

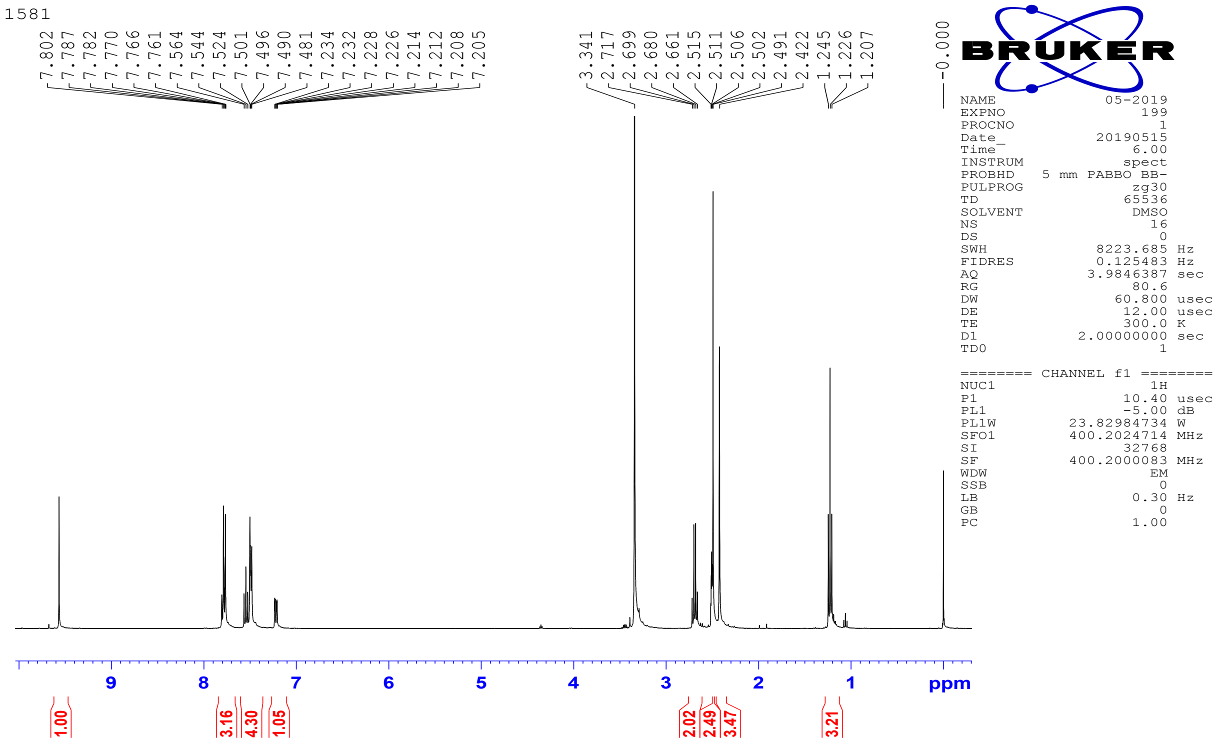

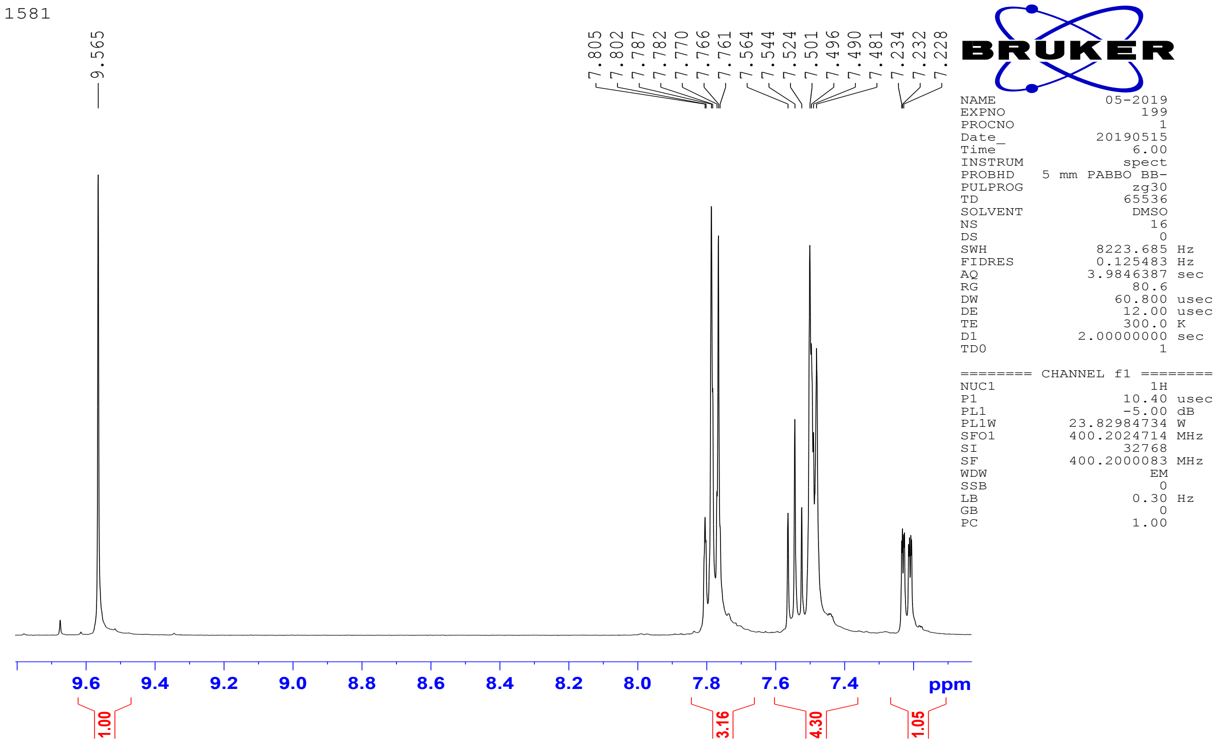

Figure S3. ^{1}H-NMR (400 MHz, DMSO) spectrum of compound **6b**.

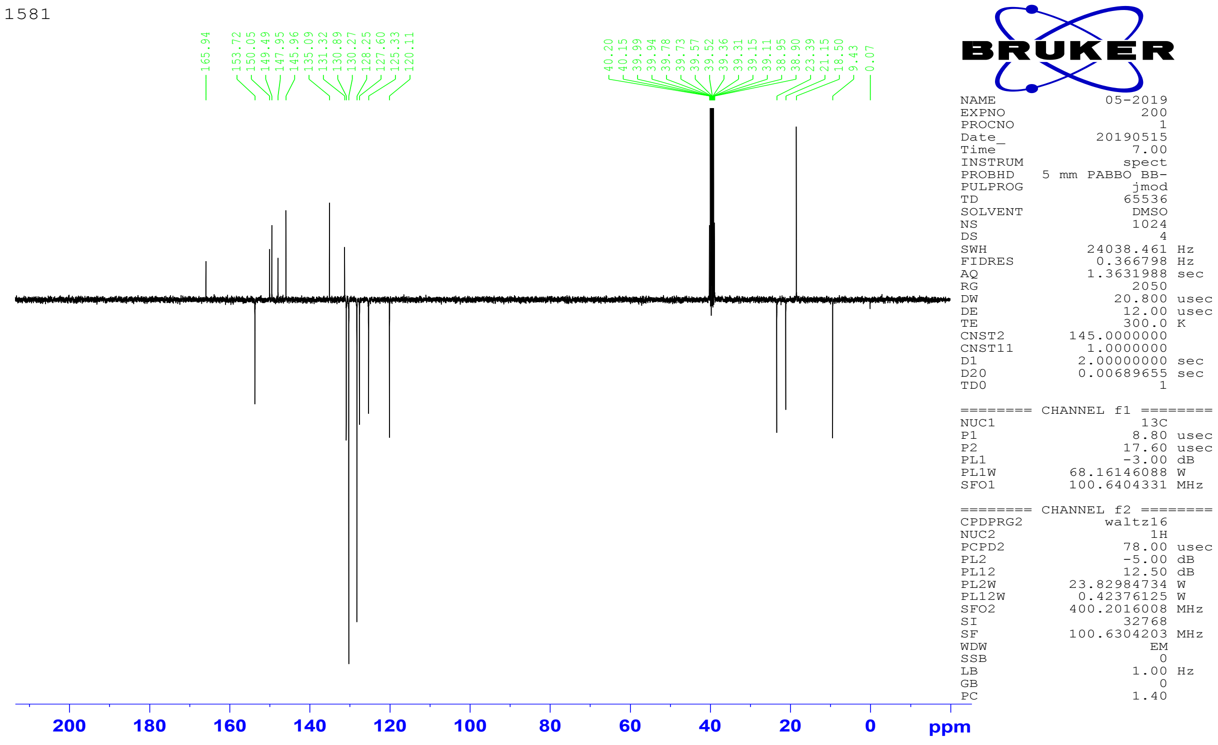

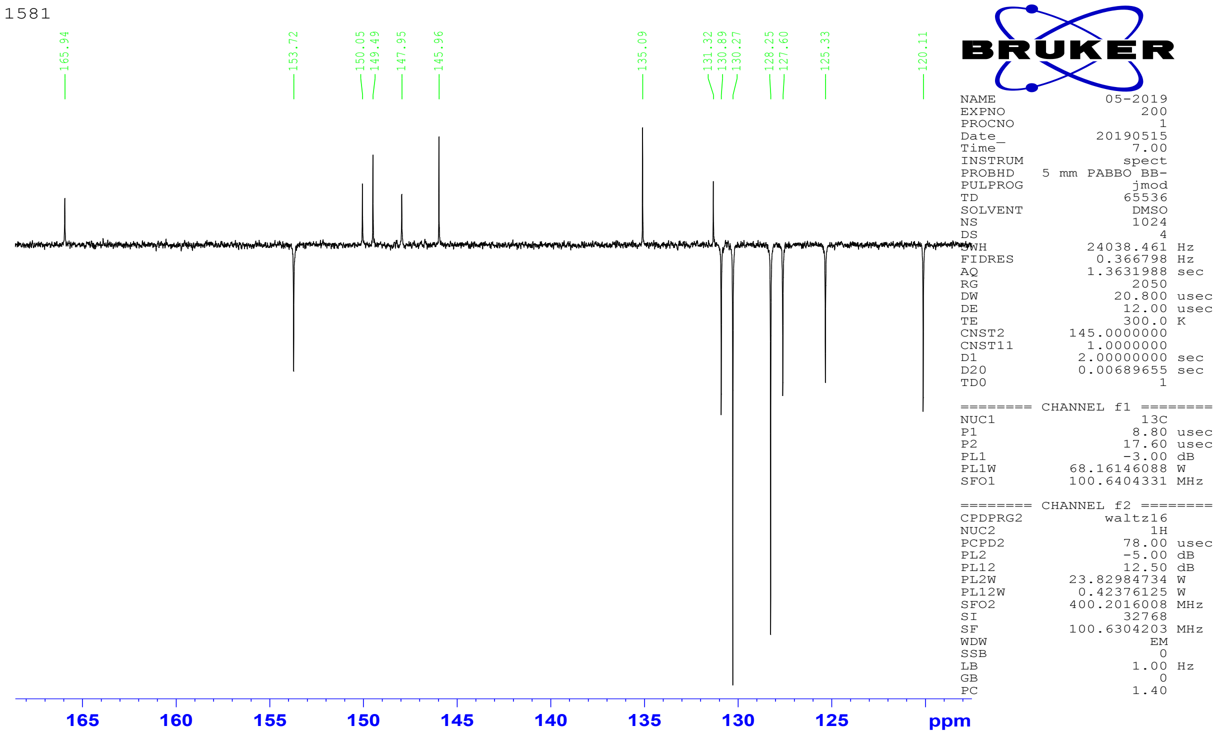

Figure S4. ^{13}C-NMR (100 MHz, DMSO) spectrum of compound **6b**.

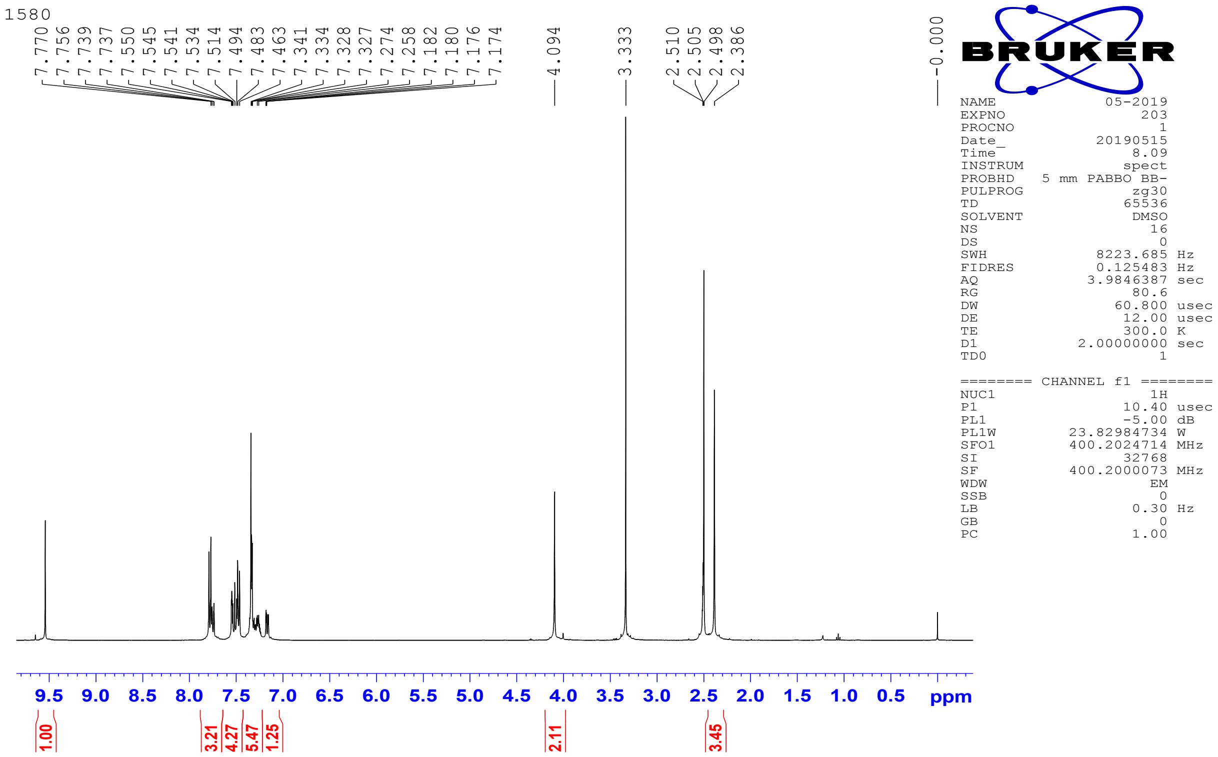

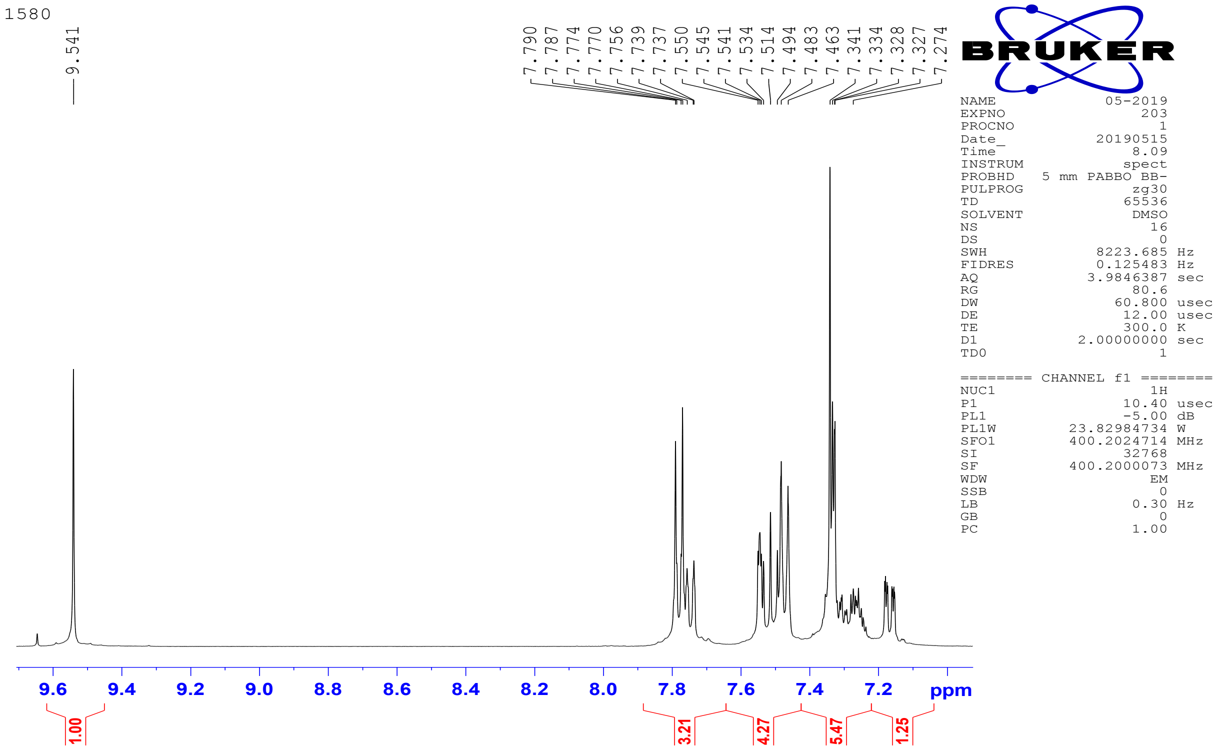

Figure S5. ^{1}H-NMR (400 MHz, DMSO) spectrum of compound **6c**.

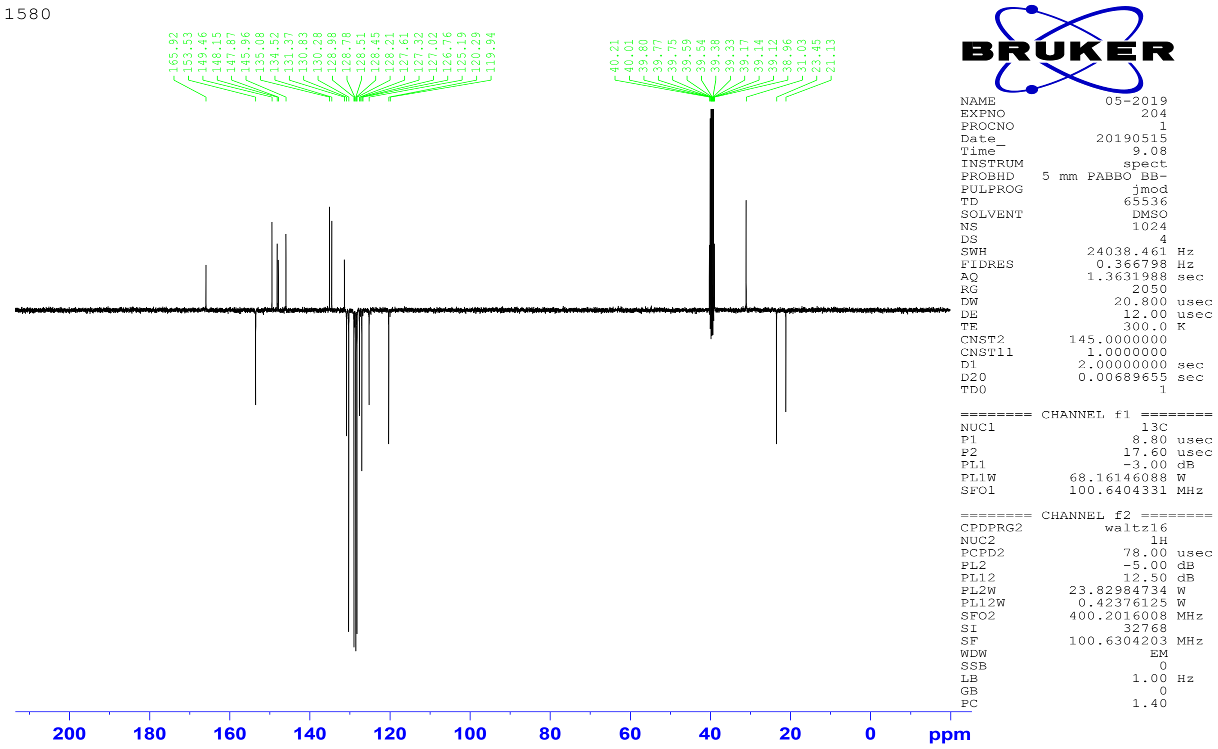

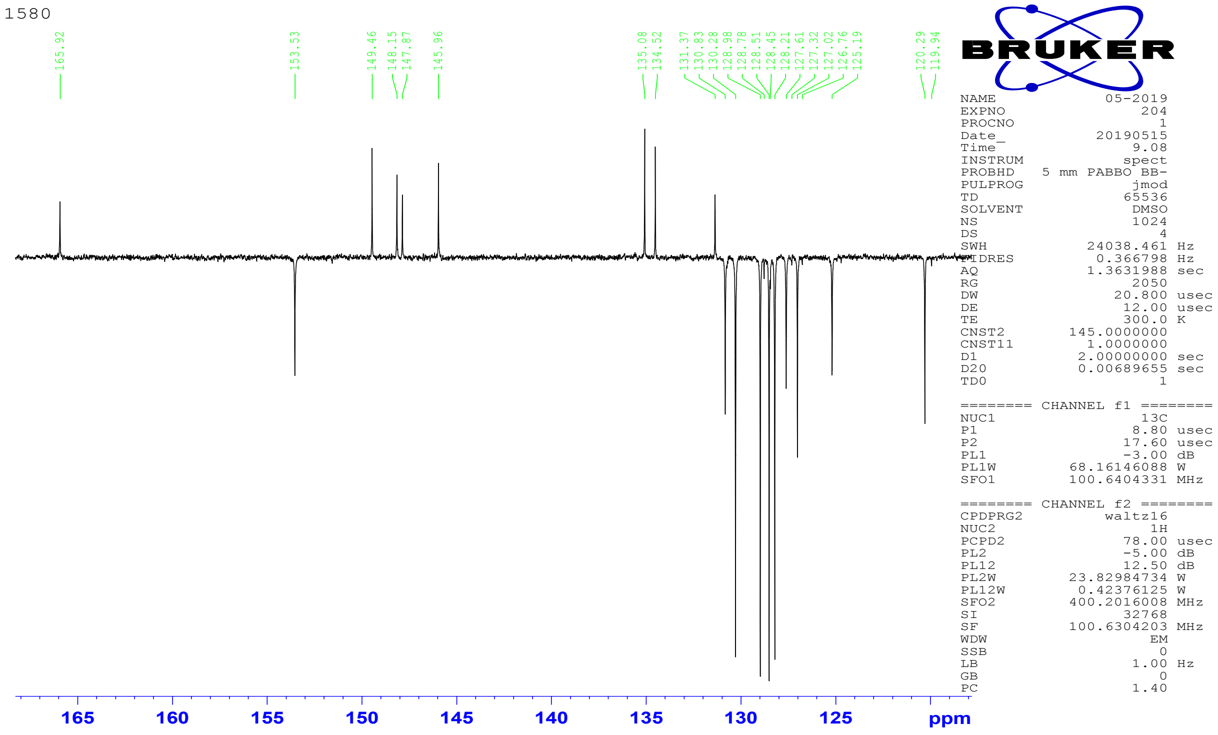

Figure S6. ^{13}C-NMR (100 MHz, DMSO) spectrum of compound **6c**.

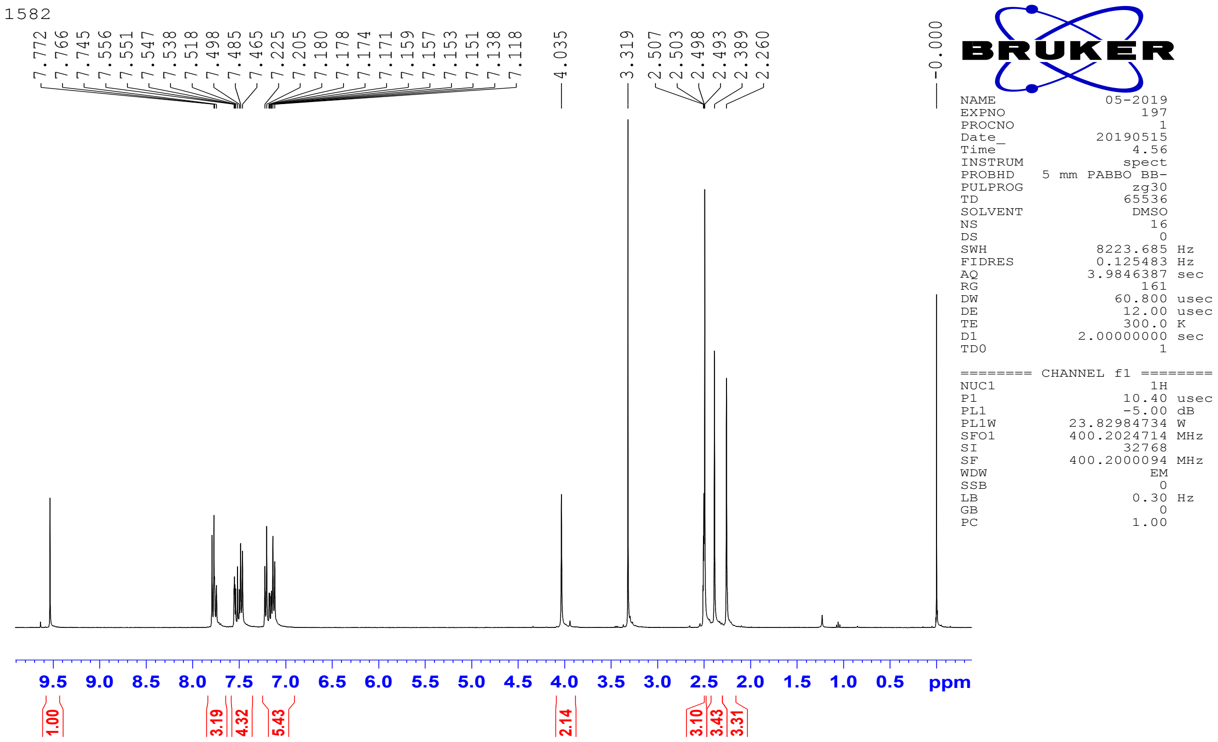

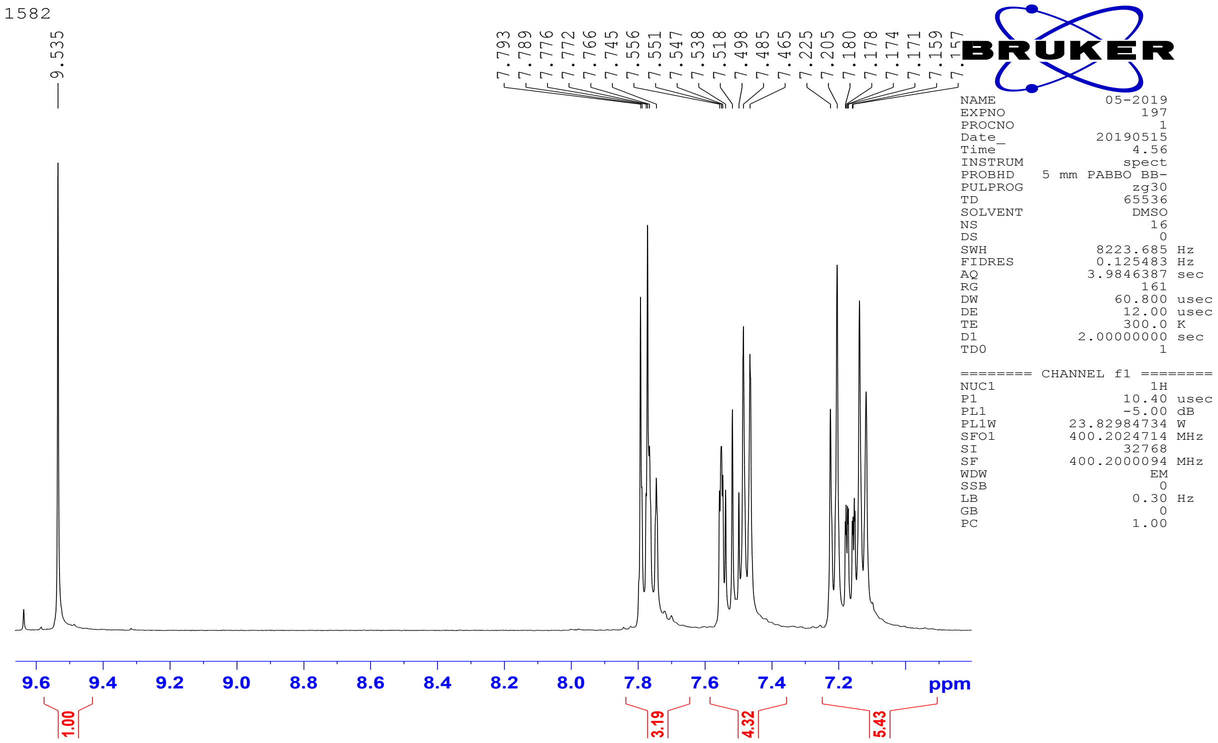

Figure S7. ^{1}H-NMR (400 MHz, DMSO) spectrum of compound **6d**.

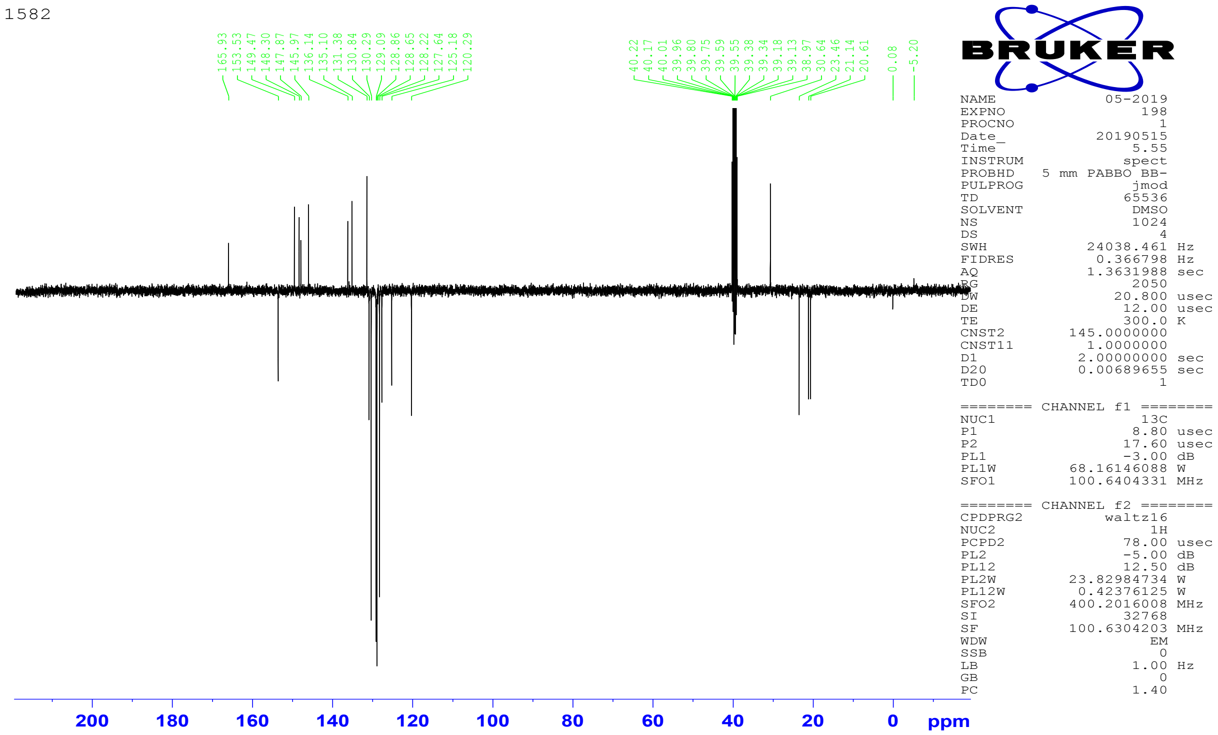

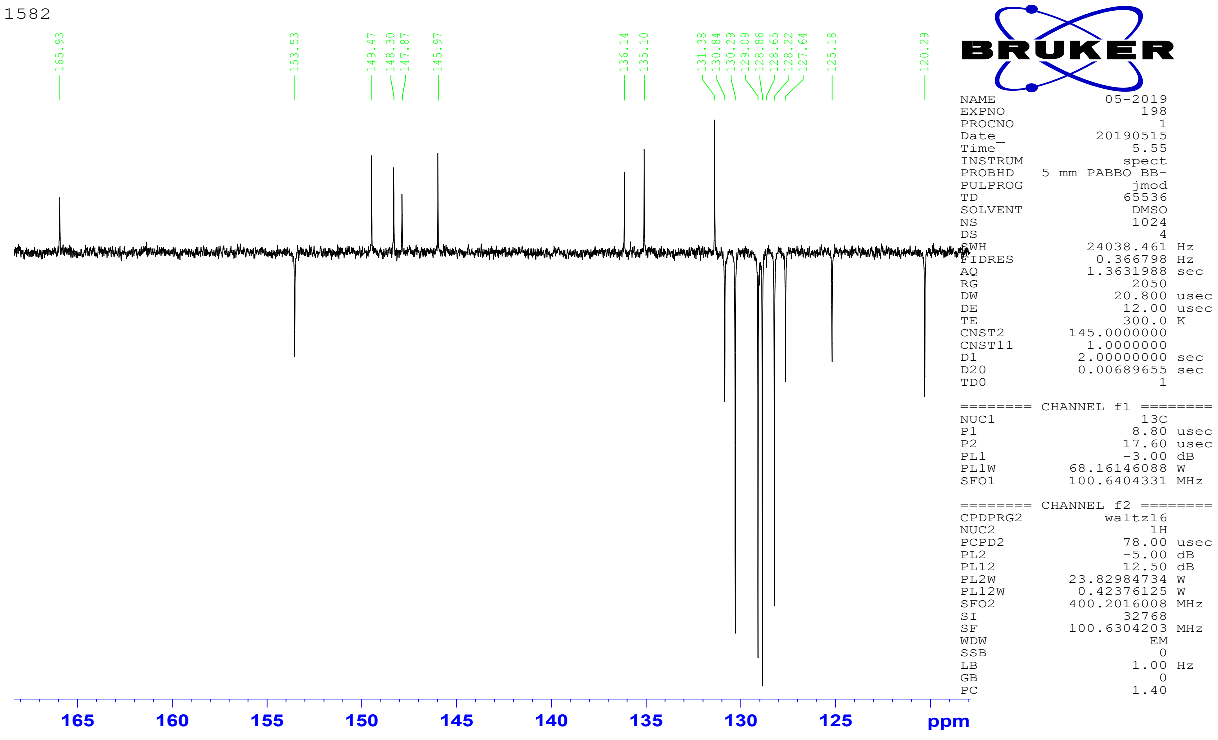

Figure S8. ^{13}C-NMR (100 MHz, DMSO) spectrum of compound **6d**.

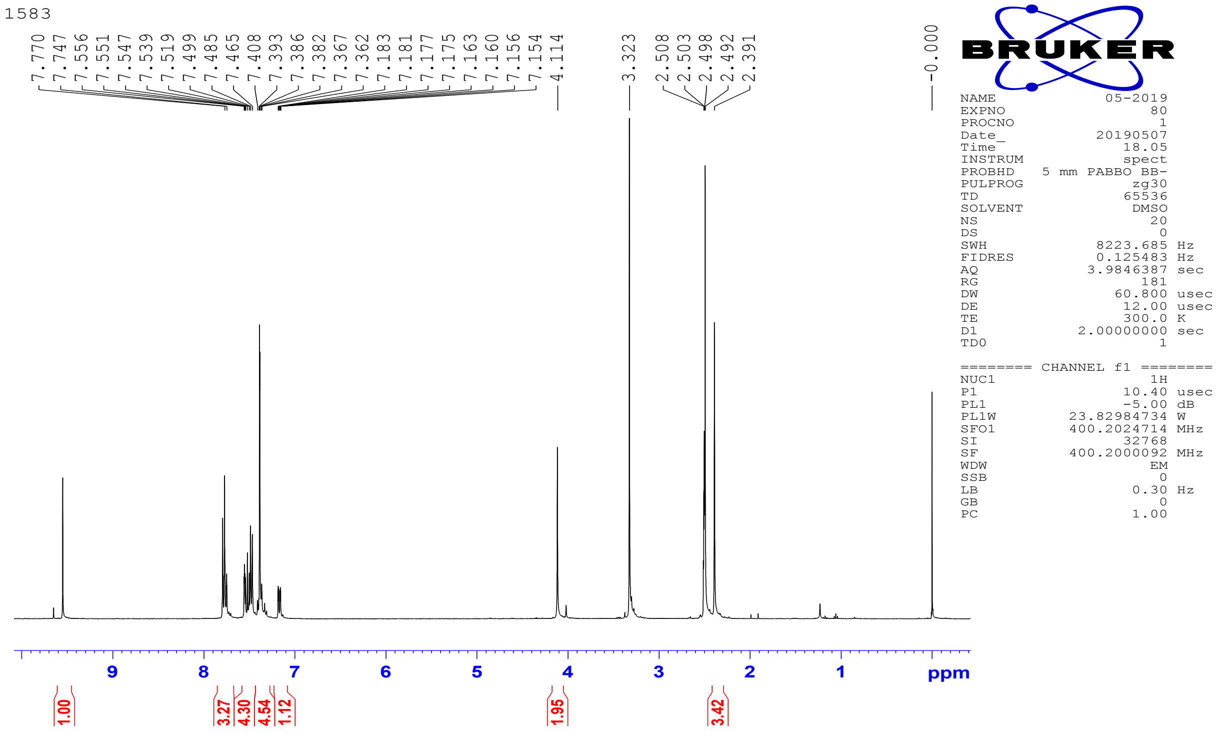

Figure S9. ^{1}H-NMR (400 MHz, DMSO) spectrum of compound **6e**.

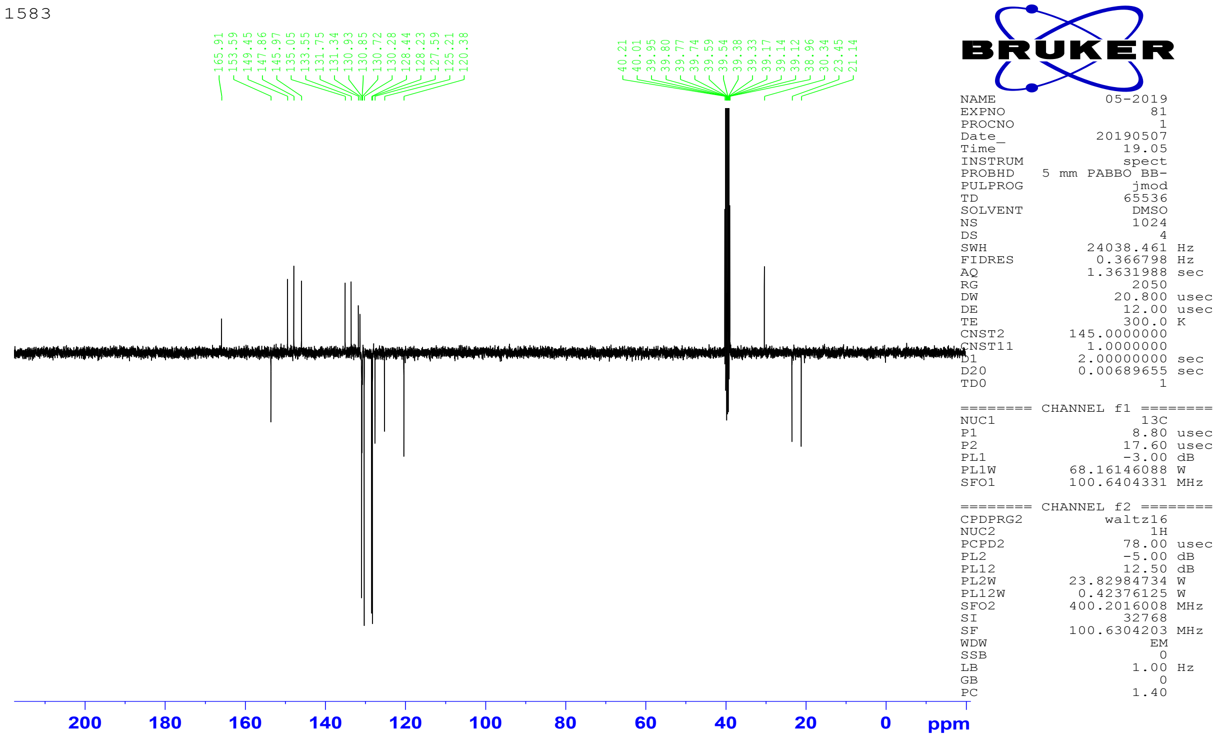

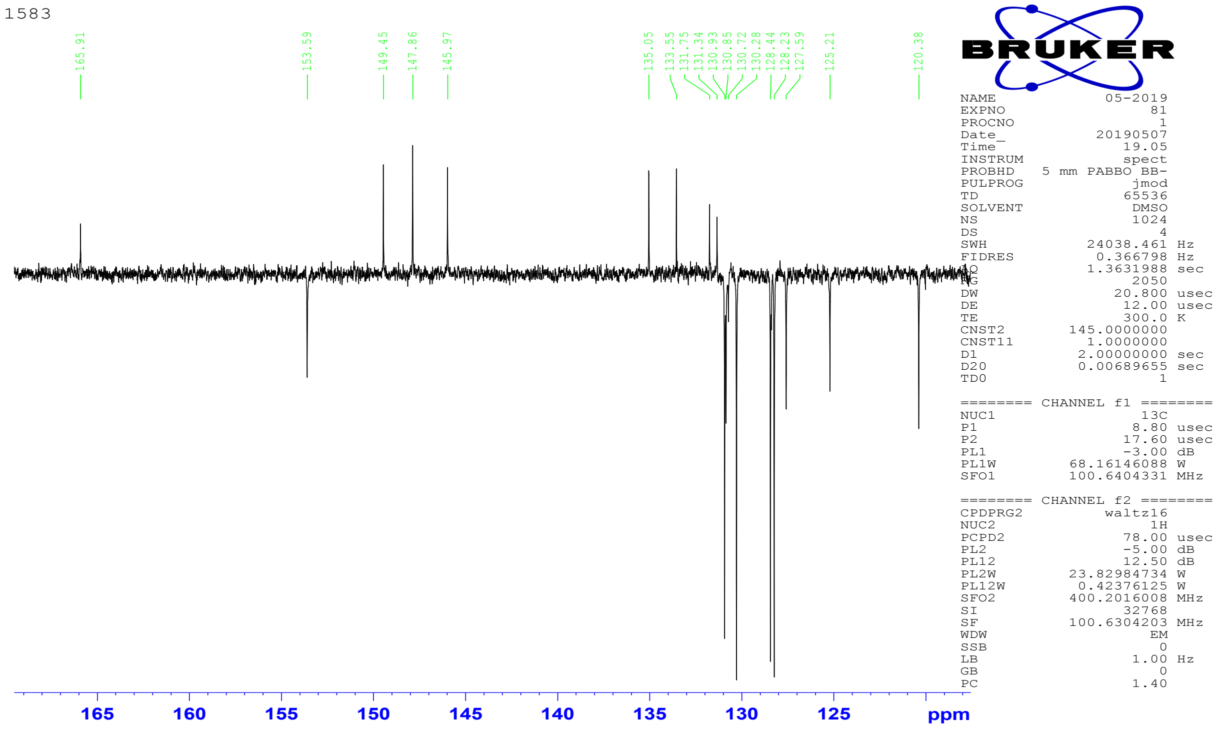

Figure S10. ^{13}C-NMR (100 MHz, DMSO) spectrum of compound **6e.**

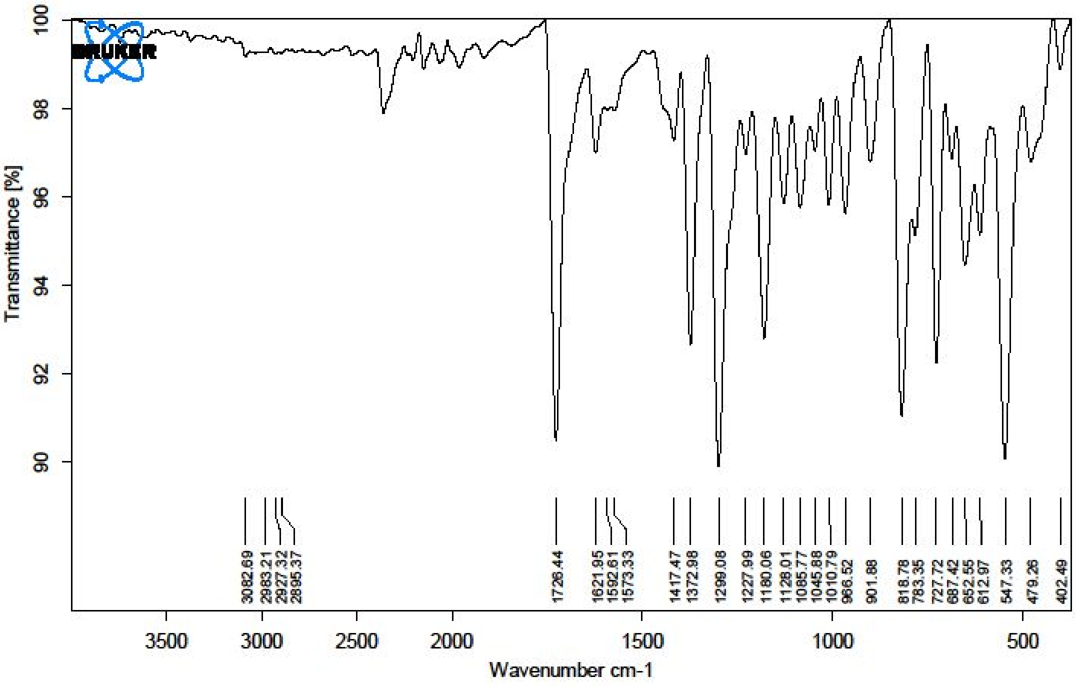

Figure S11. IR spectrum of compound **6a**.

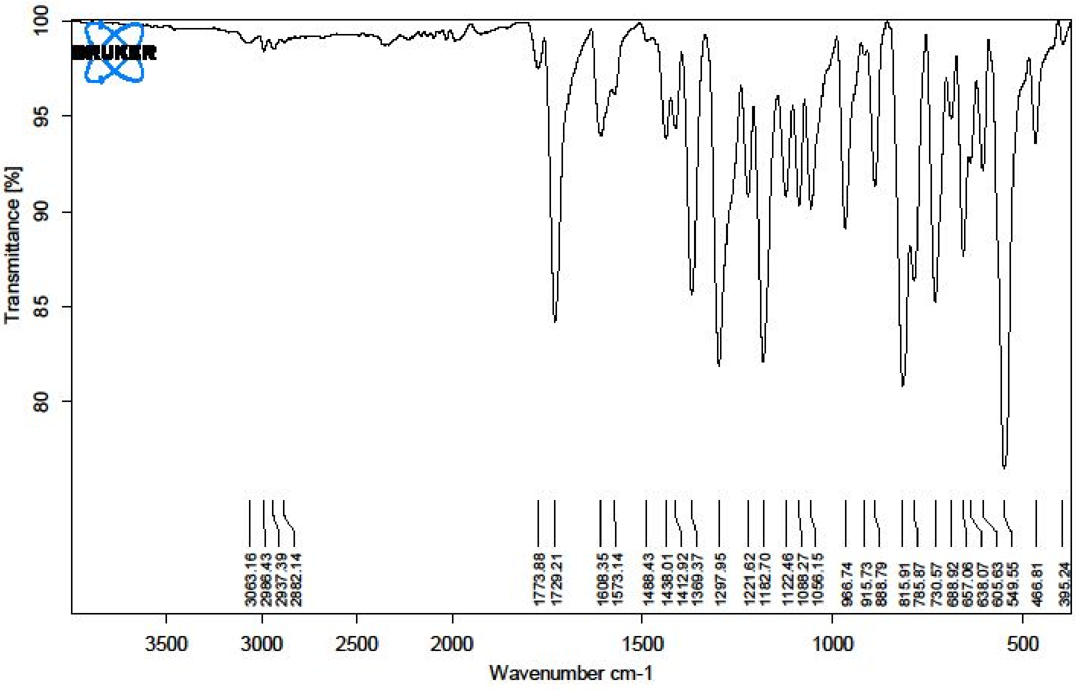

Figure S12. IR spectrum of compound **6b**.

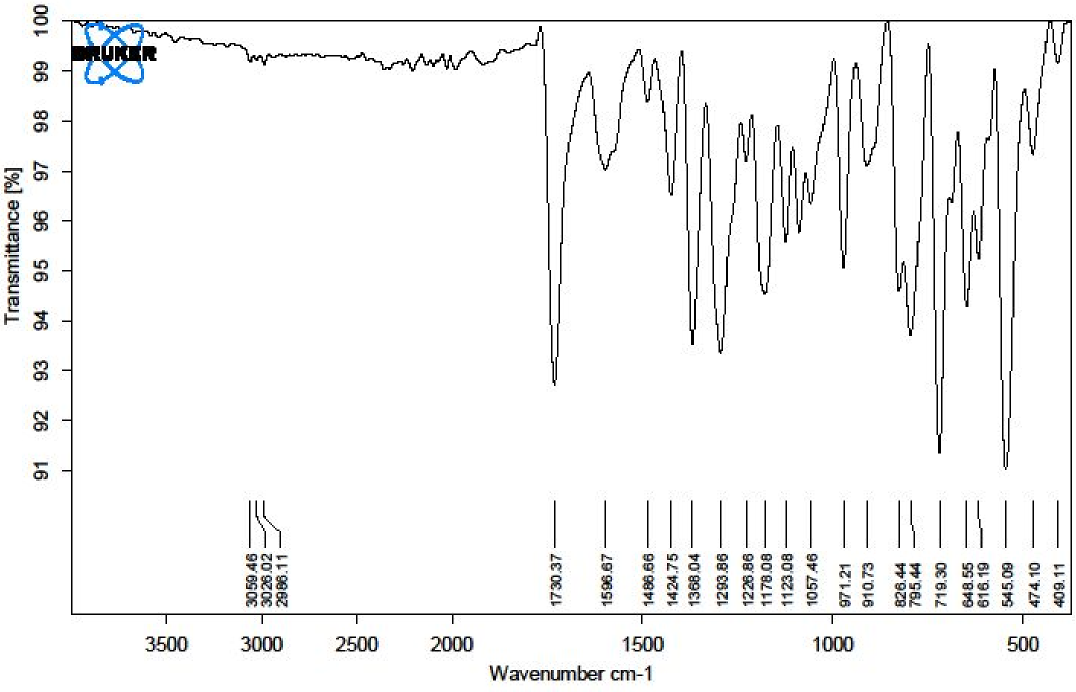

Figure S13. IR spectrum of compound **6c**.

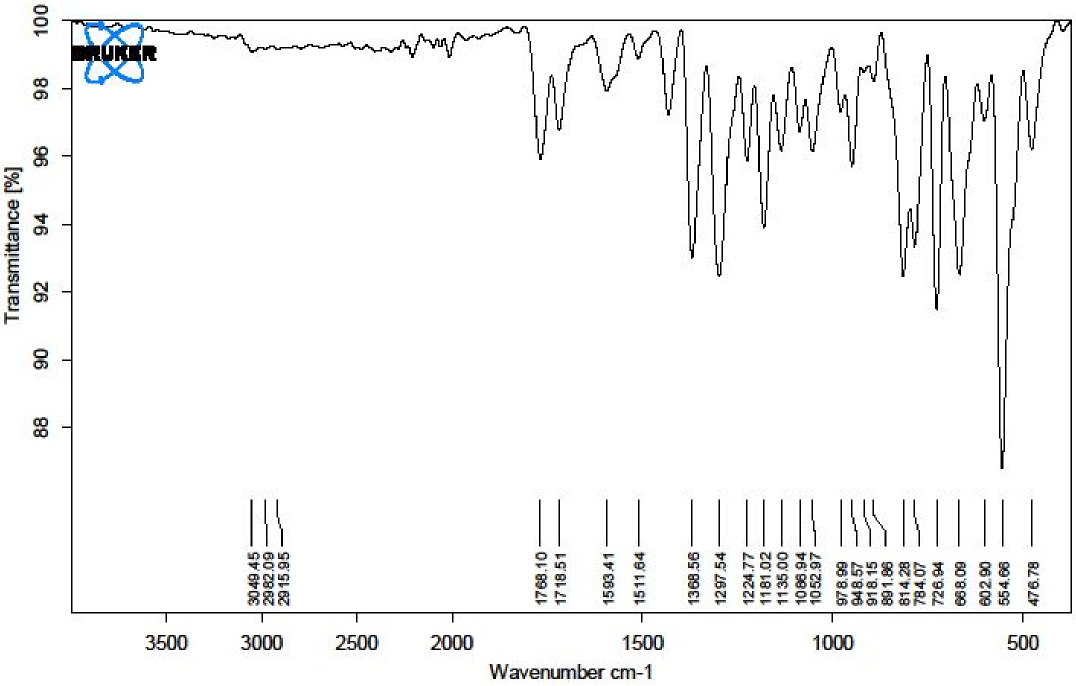

Figure S14. IR spectrum of compound **6d**.

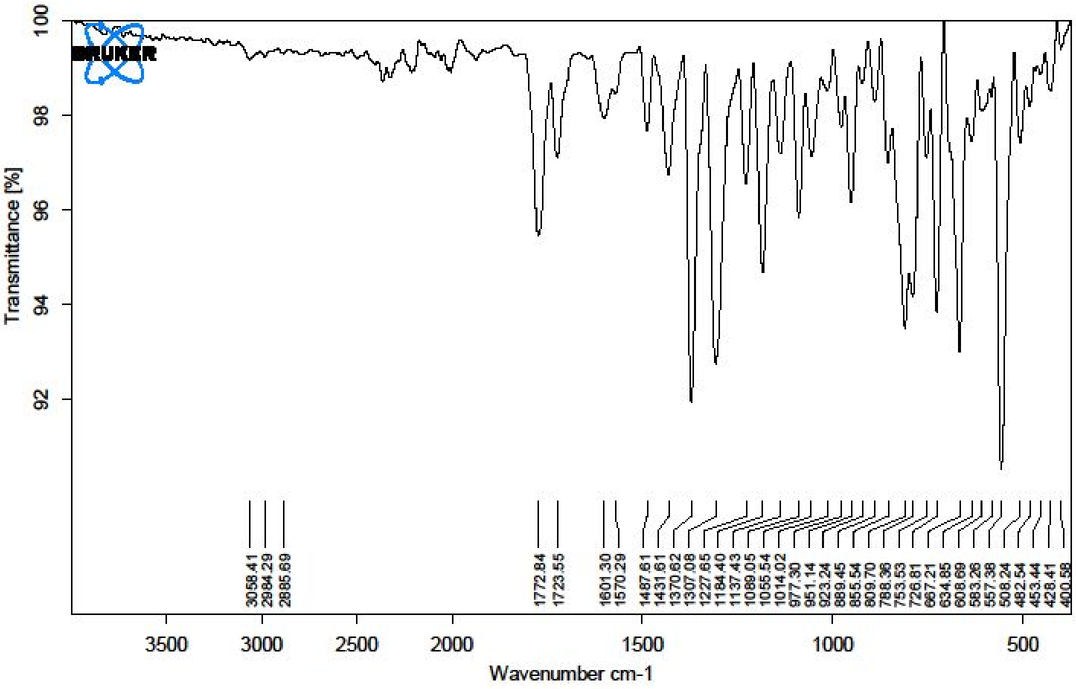

Figure S15. IR spectrum of compound **6e**.

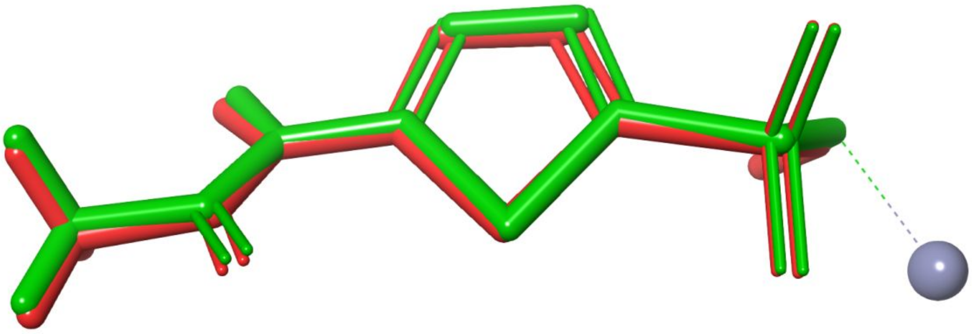

Figure S16. Docking validation for CA I (PDB ID: 1AZM) RMSD: 0.0021 Å.

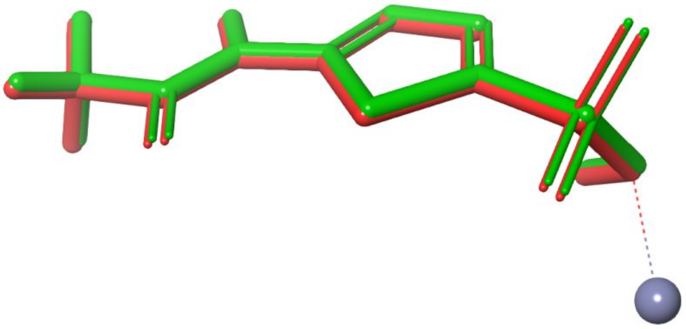

Figure S17. Docking validation for CA II (PDB ID: 3HS4) RMSD: 0.0015 Å.

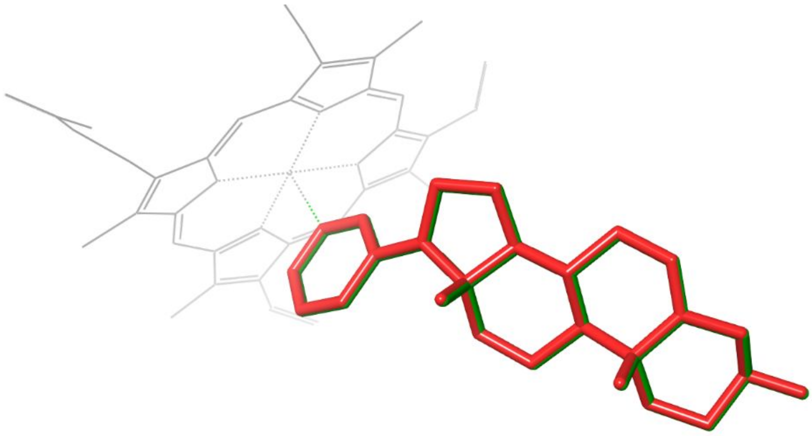

Figure S18. Docking validation for CYP17A1 (PDB ID: 3RUK) RMSD: 0.0001 Å.

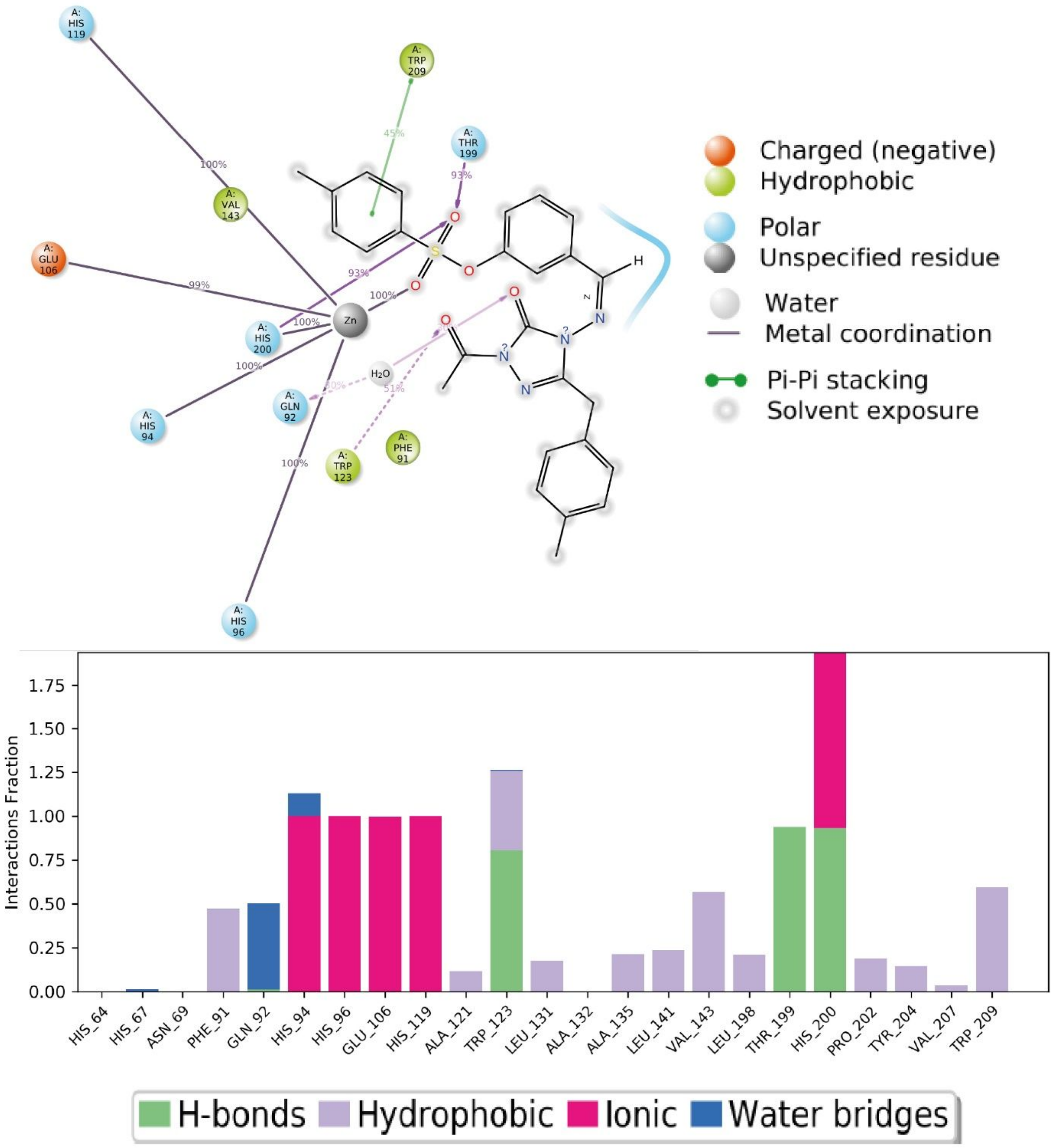

Figure S19. All interactions occurring during the MD simulation of CAI-Compound **6d**.

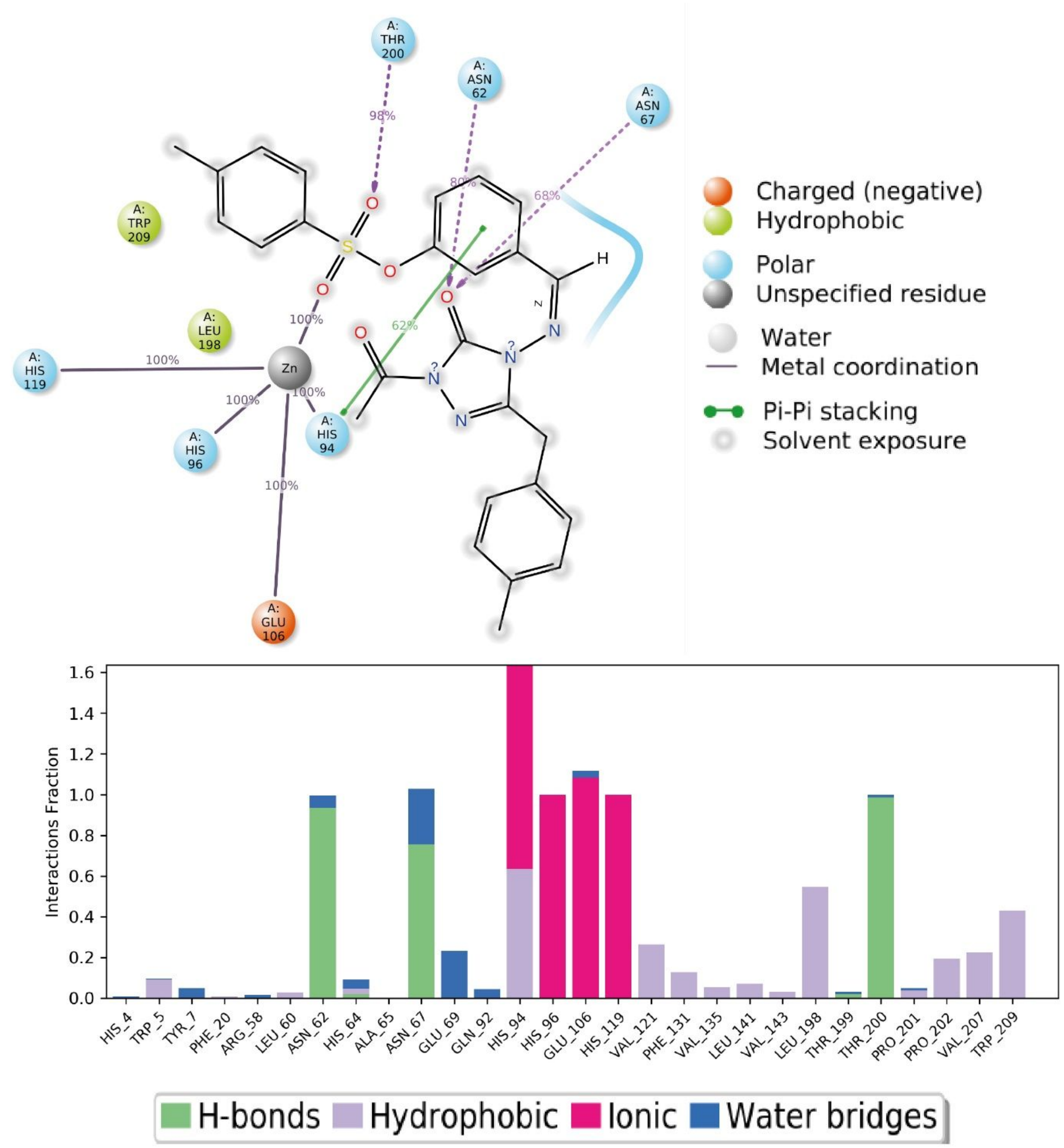

Figure S20. All interactions occurring during the MD simulation of CAII-Compound **6d**.

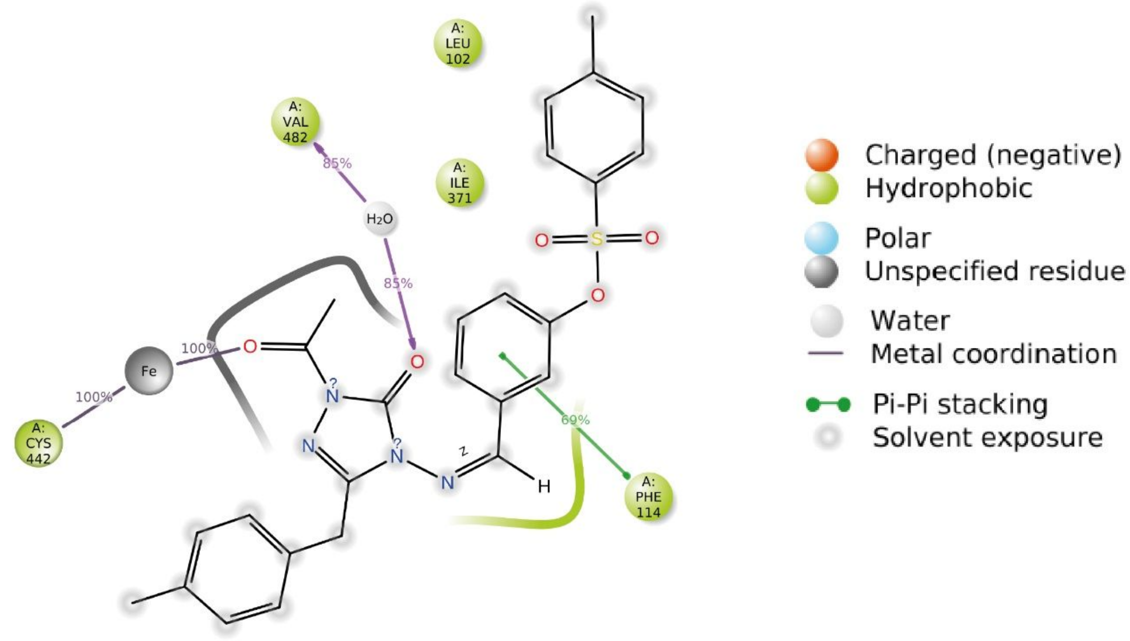

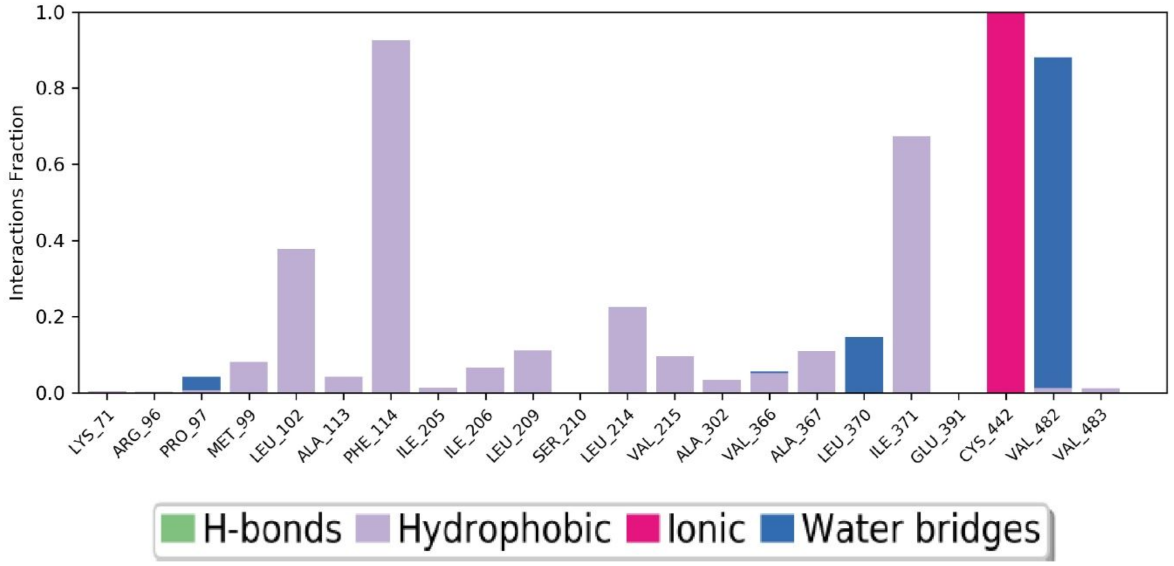

Figure S21. All interactions occurring during the MD simulation of CYP17A1-Compound **6d**.
